# Mindfulness training preserves sustained attention and resting state anticorrelation between default‐mode network and dorsolateral prefrontal cortex: A randomized controlled trial

**DOI:** 10.1002/hbm.25197

**Published:** 2020-09-24

**Authors:** Clemens C. C. Bauer, Liron Rozenkrantz, Camila Caballero, Alfonso Nieto‐Castanon, Ethan Scherer, Martin R. West, Michael Mrazek, Dawa T. Phillips, John D. E. Gabrieli, Susan Whitfield‐Gabrieli

**Affiliations:** ^1^ Department of Brain and Cognitive Sciences and McGovern Institute for Brain Research Massachusetts Institute of Technology Cambridge Massachusetts USA; ^2^ Department of Psychology Northeastern University Boston Massachusetts USA; ^3^ Department of Psychology Yale University New Haven Connecticut USA; ^4^ Department of Speech, Language and Hearing Sciences Boston University Boston Massachusetts USA; ^5^ Harvard Graduate School of Education Cambridge Massachusetts USA; ^6^ Department of Psychological and Brain Sciences University of California Santa Barbara California USA; ^7^ Empowerment Holdings Santa Barbara California USA; ^8^ International Mindfulness Teachers Association Wakefield Massachusetts USA; ^9^ MIT Integrated Learning Initiative Cambridge Massachusetts USA

**Keywords:** children, default mode network, functional connectivity, mindfulness training, resting state, sustained attention

## Abstract

Mindfulness training can enhance cognitive control, but the neural mechanisms underlying such enhancement in children are unknown. Here, we conducted a randomized controlled trial (RCT) with sixth graders (mean age 11.76 years) to examine the impact of 8 weeks of school‐based mindfulness training, relative to coding training as an active control, on sustained attention and associated resting‐state functional brain connectivity. At baseline, better performance on a sustained‐attention task correlated with greater anticorrelation between the default mode network (DMN) and right dorsolateral prefrontal cortex (DLPFC), a key node of the central executive network. Following the interventions, children in the mindfulness group preserved their sustained‐attention performance (i.e., fewer lapses of attention) and preserved DMN–DLPFC anticorrelation compared to children in the active control group, who exhibited declines in both sustained attention and DMN–DLPFC anticorrelation. Further, change in sustained‐attention performance correlated with change in DMN–DLPFC anticorrelation only within the mindfulness group. These findings provide the first causal link between mindfulness training and both sustained attention and associated neural plasticity. Administered as a part of sixth graders' school schedule, this RCT supports the beneficial effects of school‐based mindfulness training on cognitive control.

## Graphical abstract

Mindfulness training enhances cognitive control by preserving sustained‐attention performance (i.e., fewer lapses of attention) and preserving default mode network–dorsolateral prefrontal cortex (DMN–DLPFC) anticorrelation compared to children in the active control group, who exhibited declines in both sustained attention and DMN–DLPFC anticorrelation. Further, change in sustained‐attention performance correlated with change in DMN–DLPFC anticorrelation only within the mindfulness group. These findings provide the first causal link between mindfulness training and both sustained attention and associated neural plasticity.

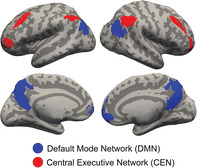

## 1 INTRODUCTION

Cognitive control, or executive function, refers to a suite of related processes by which goals or plans influence behavior. These processes include the ability to focus on a task for prolonged periods while inhibiting inappropriate responses or mind wandering (i.e., sustained attention) and the capacity to maintain goal‐relevant information in mind (i.e., working memory) (Flook et al., 2010; Geier, Garver, Terwilliger, & Luna, 2009; Sarter, Givens, & Bruno, 2001). Sustained attention is essential for learning and academic achievement (Spira & Fischel, 2005), and children's attentional skills play a significant role in their school performance (Muris, 2006), even after controlling for factors such as maternal education (Wilson, Petaja, & Mancil, 2011), family income (Duncan et al., 2008; Muris, 2006; Wilson et al., 2011), IQ (Rabiner & Coie, 2000), and behavioral problems (Duncan et al., 2008; Giannopulu, Escolano, Cusin, Citeau, & Dellatolas, 2008; Rabiner & Coie, 2000). Fostering the ability to sustain attention, therefore, ought to be helpful for a child to learn and achieve academically.

There is considerable evidence that mindfulness training enhances cognitive control in adults and children (Chiesa, Calati, & Serretti, 2011; Mak, Whittingham, Cunnington, & Boyd, 2018), but the neural mechanisms of such enhancement are unknown. Here we asked in a randomized controlled trial (RCT) whether grade‐wide mindfulness training in sixth graders would enhance sustained attention and, for the first time, assessed the underlying brain plasticity associated with mindfulness‐driven gains in sustained attention in children.

Systematic reviews of mindfulness RCT studies have reported that mindfulness training frequently improves cognitive control in both children and adults (Cásedas, Pirruccio, Vadillo, & Lupiáñez, 2019; Chiesa et al., 2011; Mak et al., 2018). A meta‐analysis of RCTs employing mindfulness‐based interventions in adults found a moderate but significant improvement of cognitive control, specifically in attention and memory (Cásedas et al., 2019). For children and adolescents, 8 out of 10 RCTs reported that mindfulness training improved cognitive control and attentional abilities (with an additional trend in a ninth study) (Britton et al., 2014; Felver, Tipsord, Morris, Racer, & Dishion, 2017; Flook et al., 2010; Lawler, Esposito, Doyle, & Gunnar, 2019; Leonard et al., 2013; Quach, Jastrowski Mano, & Alexander, 2016; Salmoirago‐Blotcher et al., 2019; Schonert‐Reichl et al., 2015; Semple, Lee, Rosa, & Miller, 2010; Sidhu, 2012).

A critical component of cognitive control is sustained attention, which involves the ability to focus on external, task‐relevant stimuli and responses, and to suppress task‐irrelevant thoughts and feelings (i.e., lapses of attention or mind wandering). These dual processes correspond to brain activations in two neural networks: the central executive network (CEN) and the default mode network (DMN). The CEN, with core nodes located in bilateral dorsolateral prefrontal cortices (DLPFCs) and bilateral parietal cortices, typically exhibits increased activation during engagement in attention‐demanding tasks (Denkova, Nomi, Uddin, & Jha, 2019; Greicius, Krasnow, Reiss, & Menon, 2003; Mason et al., 2007; Weissman, Roberts, Visscher, & Woldorff, 2006). Further, lesions to DLPFC enhance distractibility and impair attention and goal‐directed behavior (Chao & Knight, 1998; Woods & Knight, 1986). The DMN is associated with mind‐wandering and task‐irrelevant thoughts (Christoff, Gordon, Smallwood, Smith, & Schooler, 2009; Denkova et al., 2019; Fox & Raichle, 2007; Posner, Park, & Wang, 2014). Greater activations in core nodes of the DMN, namely medial prefrontal cortex (MPFC), posterior cingulate cortex (PCC), and bilateral parietal cortices, lead to reduced vigilance and increased mind wandering (Christoff et al., 2009; Hinds et al., 2013).

Thus, sustained attention requires a balance between the CEN and DMN systems, operationalized as both increased activation of the CEN and reduced activation of DMN. Lack of segregation in activations of these networks leads to failure to attend to a task: Lapses in attention followed reduced activations in attention‐related brain regions, including the CEN, along with reduced deactivations of the DMN (Weissman et al., 2006). Such functional segregation can also be measured as a negative correlation (or anticorrelation) in functional connectivity patterns between core nodes of these two networks. Indeed, studies have found enhanced anticorrelation between the DMN and DLPFC during attention‐demanding tasks (Denkova et al., 2019; Greicius et al., 2003; Piccoli et al., 2015). Further, stronger anticorrelations between the DMN and a network of task‐activated regions, including DLPFC, were associated with more consistent performance on an attention‐demanding task (Kelly et al., 2008). Together, a line of task‐based evidence demonstrates the relationship between sustained attention and DMN–CEN anticorrelation.

Resting‐state studies have also revealed both positive and negative correlations (or anticorrelations) between brain regions. The positive correlations are observed between brain regions that have often been identified as supporting orchestrated functions (e.g., bilateral frontal and parietal regions which constitute the CEN, all related to cognitive control;Fox et al., 2005; Fox & Raichle, 2007; Posner et al., 2014). Such patterns of correlations at rest indicate that these regions are integrated as a network, even in the absence of a task. Negative correlations, or anticorrelations, at rest are interpreted as segregations between networks that may be functionally competitive, such as the DMN and CEN. Indeed, an anticorrelation between DMN and CEN is observed even in the absence of task performance during resting state (Fox et al., 2005; Fox & Raichle, 2007; Posner et al., 2014), reflecting the functional segregation between these two networks. In addition, stronger DMN–CEN anticorrelation at rest has been associated with better cognitive control in adults; stronger resting‐state anticorrelation (i.e., more negatively correlated) between MPFC and DLPFC has been associated with greater working memory capacity (Hampson, Driesen, Roth, Gore, & Constable, 2010; Keller et al., 2015; Whitfield‐Gabrieli et al., 2018). In elderly adults, a weakening of DMN–CEN anticorrelation over 4 years was associated with a decline in processing speed (Ng, Lo, Lim, Chee, & Zhou, 2016). Further, clinical populations with cognitive control difficulties, such as ADHD (Hoekzema et al., 2014; Mattfeld et al., 2014) and schizophrenia (Whitfield‐Gabrieli et al., 2009), have reduced resting‐state DMN–CEN anticorrelations.

Here, we asked whether mindfulness training in children would enhance sustained attention and whether such an enhancement would be related to brain plasticity in the relations between the DMN and CEN. Behavioral enhancement and brain plasticity seemed plausible because mindfulness entails a continuous practice in cultivating attention to the present moment while continuously rejecting distractions. Using an RCT design, we were able to compare the effect of mindfulness training versus computer coding training (i.e., active control) on neurocognitive processes. The intervention was grade‐wide (i.e., all sixth graders in the school participated) and included 99 children (mean age 11.76 years). Results reported here come from the subset of children whose families opted to participate in a neuroimaging visit at pre‐ and post‐intervention, which was approximately one‐third (34.3%) of all children enrolled in the full‐scale RCT (all families were invited).

In the present study, we measured sustained attention by performance on the Sustained Attention to Response Task (SART) before and after the interventions. This task requires the participant to press a button when presented with any digit (0–9, Go trials), except for the rarely presented “3” that appears on only 5% of trials (No‐Go trials). As the task lasts approximately 15 min, it requires sustained attention for a tedious task over a long period. Performance on the Go trials provides a measure of sustained attention, whereas performance on the No‐Go trials provides a measure of response inhibition (Allan Cheyne, Solman, Carriere, & Smilek, 2009; Robertson, Manly, Andrade, Baddeley, & Yiend, 1997a; Smallwood, 2013). We measured resting‐state functional connectivity (rsFC) before and after the intervention.

We tested three main hypotheses. First, we asked whether the initial ability to sustain attention on the SART was associated with rsFC anticorrelation between DMN and CEN networks. This would be the first study to probe the link between sustained attention and patterns of rsFC in children. Second, we asked whether mindfulness training would enhance sustained attention on the SART relative to coding training. Third, we asked whether mindfulness training, relative to coding training, would strengthen DMN–CEN anticorrelation. Further, to directly associate behavioral and brain plasticity, we examined whether pre–post intervention changes in sustained attention and in DMN–CEN anticorrelation would be correlated among the children who received the mindfulness training. The RCT design of the study could provide novel causal evidence about the effect of mindfulness training on sustained attention and its underlying brain plasticity.

## 2 METHODS

### 2.1 Participants and randomization procedures

Ninety‐nine sixth graders at the Boston Collegiate Charter School, a public charter school in Dorchester, MA, were randomly assigned to either a mindfulness training group or a coding training group during which they learned about computer coding. These interventions lasted for 8 weeks and took place during the last class period of their school‐day schedule, which is typically reserved for miscellaneous school‐related activities. All students were invited to participate in the brain imaging protocol at the Massachusetts Institute of Technology, of whom 40 students volunteered and completed the imaging protocol. Participant characteristics were that 70% were female; 47.5% had ever been on the free/reduced price lunch (FRPL) program for low‐income families; and 10% were Hispanic, 32.5% were African American, 52.5% were White, and 5% other or multiple racial identities (see Table 1).

**TABLE 1:** Pre‐intervention participant characteristics (*N* = 40)

Variable	Mean	*SD*
Age (years)	11.76	.40
Gender	12 males, 28 females	
Race/ethnicity	4 Hispanic, 13 African American 21 white, 2 other/multiple racial identity	
Handedness	33 right‐handed, 7 left‐handed	
BMI (kg/m^2^)	22.73	5.25
FRPL program	19	
WASI IQ	98.1	9.59

Abbreviations: BMI, body mass index; FRPL, free‐ and reduced‐price lunch; *SD*, standard deviation; WASI IQ, Wechsler Abbreviated Scales of Intelligence for IQ.

Pre‐intervention measures included the Wechsler Abbreviated Scales of Intelligence for IQ [WASI, Wechsler, 1999] and the Edinburgh assessment of handedness (Oldfield, 1971) administered prior to randomization. For the randomization process, we stratified on indicator variables of whether a student participated in the imaging protocol and their handedness. We ran 1,000 randomizations and calculated the Mahalanobis distance between the mindfulness training and coding training group in order to create a single multivariate distance metric for the following student characteristics: sex, age, race and ethnicity, special education, FRPL, and prior performance on state standardized test scores (Morgan & Rubin, 2012). We selected the randomization combination that minimized the Mahalanobis Distance to further reduce omitted variable biases along with the RCT design; this approach has been increasingly used by other RCTs so as to equate randomized groups on multiple dimensions (Morgan & Rubin, 2012).

Forty children completed the baseline (pre‐intervention) behavioral assessments and imaging protocol (Table 1). Thirty‐one children were included (15 in the mindfulness training, 16 in the coding training) after removing participants due to scanning contraindications (i.e., getting braces), excessive movement, and missing data (see Section 2.11.2 and Table 2). Four students were left‐handed (one in the mindfulness training, three in the coding training). The study complied with the 1975 Declaration of Helsinki and was approved by the Massachusetts Institute of Technology Committee on the Use of Humans as Experimental Subjects. Parents gave written informed consent for their children to participate in the study and children gave written informed assent for their participation. Families were compensated with gift cards for their participation.

**TABLE 2:** Pre‐intervention characteristics of participants included in pre–post training (*N* = 31)^a^

Characteristic	Mindfulness group *N* = 15	Coding group *N* = 16	Difference statistic
Age (mean years (*SD*))	12.07 (.47)	11.94 (.37)	*t*(30) = .79, *p* = .43
Gender			*x* ^2^(1) = 2.88, *p* = .08^b^
Male	2	7	
Female	14	8	
Handedness			*x* ^2^(1) = .28, *p* = .59^b^
Right	15	13	
Left	1	3	
BMI (kg/m^2^)	22.25 (5.27)	22.36 (5.80)	*t*(30) = .58, *p* = .56
SART			
Go‐Accuracy	.90 (.09)	.90 (.07)	*t*(30) = .21, *p* = .83
No‐Go‐Accuracy	.11(.08)	.12 (.10)	*t*(30) = .91, *p* = .36
WASI IQ	99.69 (10.2)	100.0 (6.4)	*t*(30) = .52, *p* = .60

Abbreviations: BMI, body mass index; FRPL, free‐ and reduced‐price lunch; SART, Sustained Attention to Response Task; *SD*, standard deviation; WASI IQ, Wechsler Abbreviated Scales of Intelligence for IQ.

^a^ Two participants were MRI incompatible at post‐intervention, seven had excess movement during imaging (see Section 2). Results are presented as mean and (*SD*).

^b^ Chi‐square statistic with Yates correction.

### 2.2 Mindfulness training group

A school‐based mindfulness training program was adapted by Calmer Choice (n.d.) to be appropriate for middle school children. The mindfulness curriculum aimed to train skills related to physical and mental strategies of focused‐attention and changing students' mindsets about their stress, negative affect, and other beliefs and attitudes (Dweck, 2006; Lutz, Slagter, Dunne, & Davidson, 2008; Yeager & Dweck, 2012).

Each class incorporated 5–15 min of mindfulness exercises requiring participants to attend to some aspect of present‐moment experience (e.g., sensations of breathing, sensations of the body, sounds in and out of the room, thoughts, or emotions) and to refocus attention on the present moment when the mind engages with cognitive processes (e.g., thinking about the past or future) or meta‐cognitive processes (e.g., appraising thoughts). Participants shared their experiences with the class and received personalized feedback from the instructor. Class content was designed to provide a clear set of strategies for practicing mindfulness as well as foster a conceptual understanding of mindfulness practice. Classes focused on (a) sitting in an upright posture with backs straight and gaze lowered or eyes closed, (b) distinguishing between naturally arising thoughts and elaborated thinking, (c) minimizing the distracting quality of past and future concerns by reframing them as mental projections occurring in the present, (d) using the breath as an anchor for attention during mindfulness exercises, (e) repeatedly counting up to five consecutive exhalations, and (f) allowing the mind to rest naturally rather than trying to suppress the occurrence of thoughts. The course lasted 8 weeks during which students met four times per week for 45‐min classes, totaling approximately 24 hr of group practice and instruction by the end of the intervention. The three trained instructors who led the intervention each had practical knowledge and experience in mindful awareness, as well as teaching mindfulness to children.

### 2.3 Coding training group

The SCRATCH (n.d.) computer programming curriculum was adapted to match the time commitment and novel engagement of the mindfulness intervention curriculum. The SCRATCH curriculum was designed to train skills of creative thinking, systematic reasoning, and collaborative work. The course met at the same time as the intervention group and also totaled approximately 24 hr of group practice and instruction by the end of the 8 weeks.

SCRATCH is a programming language and an online community where students program and share interactive media such as stories, games, and animations among them and with people from all over the world. Each class introduced step‐by‐step simple mathematical and computational ideas that were built into the SCRATCH experience in the first 15 min of the class. Students then applied the new knowledge to advance the creation of their individual programs in SCRATCH, thus applying core computational concepts such as iteration, conditionals, coordinates, variable random numbers, and so forth. Students were encouraged to share their experiences and their creative thoughts with the class and received personalized feedback from the instructor. Participants were also encouraged to work collaboratively and reason systematically. The two trained instructors who led the intervention each had practical knowledge and experience with the SCRATCH curriculum, and working with children.

### 2.4 Sustained Attention to Response Task

**FIGURE 1:**
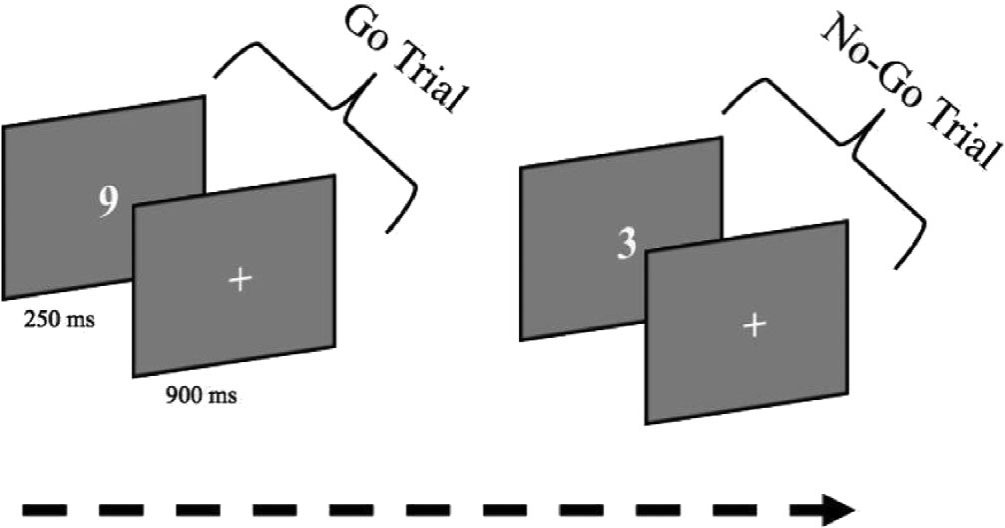
The Sustained‐Attention‐to‐Response Task (SART). Participants viewed a continuous string of single digits and were instructed to press the spacebar to all digits except 3 (“Go” trials) while withholding response to any 3 (“No‐Go”) trials). The total time for the task was ~15 min with two series and a total number of 560 individual digits (5% were targets)

We measured attentional characteristics through the SART (Robertson, Manly, Andrade, Baddeley, & Yiend, 1997b; Figure 1). The SART is a Go/No‐Go task with a high probability of “Go” signals. The SART paradigm was programmed using PsychoPy (Peirce, 2007), a python library for conducting psychological experiments. Participants were instructed to withhold responses (i.e., not pressing space bar) for the number 3 (target: “No‐Go”) and to respond quickly for all other numbers (nontargets: “Go”). Participants were instructed to respond both accurately and quickly. Participants could respond either during the stimulus display or during the intertrial interval (ITI). Participants performed a practice block consisting of 172 target and nontarget trials, immediately followed by the experimental session consisting of 2 series of 280 individual digits (28 of which were targets or 5%) for 250 ms each with an ITI of 900 ms between each digit. Trial order was pseudorandomized so that target trials were always separated by at least two nontarget trials. Participants had the option of an undefined break (not exceeding 5 min) before starting the second series. The task took approximately 15 min. to complete.

### 2.5 Attentional performance variables

The primary outcomes of the SART were accuracy on “Go” trials (nontargets) and “No‐Go” trials (targets). Accuracy on “Go” trials (hereafter: Go‐Accuracy) is an index of sustained attention (Allan Cheyne et al., 2009; Cheyne, Carriere, Solman, & Smilek, 2011) and was calculated as the percentage of correct responses (i.e., pressing for numbers 0–9 except for 3) out of all “Go” trials possible. Accuracy on “No‐Go” trials (hereafter: No‐Go‐Accuracy) is an index of response inhibition (correct withholding of a response) (McVay & Kane, 2009; Smallwood, 2013) and was calculated as the percentage of correctly withheld responses to the number 3. Speed of response was evaluated with both average RT and the intraindividual coefficient of variation (ICV). RTs below 100 ms were removed from analysis, with average RT including responses to correct trials only. ICV was calculated by dividing the *SD* of an individual's RTs by their mean RT for correct trials, with trials under 100 ms also removed. Greater ICV reflects a more variable response speed and has been implicated as a marker of off‐task thinking (Bastian & Sackur, 2013).

### 2.6 Student acceptability of interventions

We assessed student acceptability for both mindfulness and the coding training through post‐intervention surveys (Bluth et al., 2016; Britton et al., 2014; Finucane & Mercer, 2006). Four 5‐point Likert‐scale questions asked students to assess (a) their overall rating of the class, (b) the amount of work they had to do, (c) the degree of active participation, and (d) how much practical knowledge they learned.

### 2.7 Procedures and blinding

The SART was administered immediately pre‐ and post‐intervention. In addition, student acceptability surveys were collected at the end of each intervention. At each time point, trained researchers met with students in their respective homeroom classes during the school day to complete the SART in one session. The SART was administered at students' original classes (before randomization) to ensure blinding of group assignment to testers. Students and teachers were instructed to not reveal group assignment. Pre‐ and post‐intervention MRI protocols were collected before and after the end of the intervention for all participants. Pre‐intervention MRI protocols were also administered before randomization to ensure blinding of group assignment to testers. MRI technicians and researchers were blind to group assignment at all times and participants were explicitly told not to reveal group assignment at any point.

### 2.8 Mock scan session

Before the first MRI scan, all participants completed a mock‐scanner training session. Participants watched a cartoon movie in the mock scanner while their head motion was monitored. The movie would stop temporarily if their head moved more than 3 mm and resumed once no movement was detected. Recordings of the actual scanner sounds were played in the mock scanner during the training to acclimate participants to the scanner experience ahead of time. The mock scan session lasted about 30 min for each child.

### 2.9 MRI acquisition

At both neuroimaging sessions, participants underwent a 6‐min resting state scan where they were instructed to passively view a fixation cross during the scan period and not to close their eyes, sleep or engage in any mindfulness or other exercises for relaxation practices. Specific instructions were “Keep your eyes open, relax, try not to move and try to stay awake.” All scans were acquired using a 3 T Trio MR System with a 32‐channel, phased‐array head coil (Siemens Healthcare, Erlangen, Germany). Resting‐state functional magnetic resonance imaging (rs‐fMRI) was acquired using a gradient‐echo, echo‐planar imaging pulse sequence (EPI) with prospective acquisition correction (PACE) for motion (Thesen, Heid, Mueller, & Schad, 2000) with imaging parameters: repetition time (TR) = 2.2 s, echo time (TE) = 30 ms, flip angle = 90°, voxel size = 3.5 × 3.5 × 3.5, number of slices = 33, and slice gap = 10%. Online PACE was applied to the EPI sequence. PACE tracks the head of the subject and updates the position of the field‐of‐view and slice alignment during acquisition. The parameters for each time point are updated based on motion correction parameters calculated from the previous two time points. Five dummy scans were included at the start of the sequence. Additional structural scans were acquired using a three‐dimensional T1‐weighted MP‐RAGE pulse sequence with a voxel resolution of 1 mm^3^; flip angle = 7°; TE = 1.61 ms; inversion time (TI) = 1,200 ms; and TR = 2,530 ms.

### 2.10 Behavioral analyses

#### 2.10.1 Post‐intervention student acceptability ratings

Statistical tests for student acceptability scores for both mindfulness and coding groups were conducted using R Studio version 1.0.136 with R version 3.6.0 (R‐Project. R Core Team, 2014). Two‐sample *t* tests between the groups were used to assess (a) the overall rating of the class, (b) amount of work they had to do, (c) degree of active participation, and (d) how much practical knowledge they learned. Statistical significance level was set at .05.

#### 2.10.2 Effect of training on Go‐Accuracy

Statistical tests for Go‐Accuracy were conducted using R Studio version 1.0.136 with R version 3.6.0 (R‐Project. R Core Team, 2014). Regression analysis was used to assess the causal impact of mindfulness training. The model regressed Go‐Accuracy outcomes on intervention group assignment (1 for mindfulness training, 0 for coding training) taking into account pre‐intervention performance as covariate. We use heteroscedasticity‐consistent standard errors for all models. Statistical significance level was set at .05.

### 2.11 rs‐fMRI data analysis experimental design

Primary neuroimaging analysis was restricted to the DMN and CEN as a priori networks of interest based on the frequent involvement of these networks in cognitive control. First, we examined the relation of DMN–CEN anticorrelation to baseline variation in SART Go‐Accuracy. Second, we examined how the anticorrelation changed as a consequence of mindfulness training versus coding training, and whether this change was related to changes in SART Go‐Accuracy.

#### 2.11.1 Preprocessing

Data preprocessing was done using SPM12 (Friston, 2007), which for the resting state scans included motion correction, slice timing correction, normalization with respect to the EPI template (sampling size was matched to the native 2‐mm isotropic resolution) provided by SPM, and 8‐mm Gaussian smoothing. Structural scan was normalized with respect to SPM's T1 template. Finally, image segmentation was carried out on the T1‐weighted images to yield gray matter, white matter (WM), and cerebrospinal fluid (CSF) masks in normalized space (Ashburner & Friston, 2005). Additional preprocessing steps were carried out using the CONN toolbox version 19.d (Whitfield‐Gabrieli & Nieto‐Castanon, 2012). This included denoising using a Compcor (anatomical component‐based noise correction method) (Behzadi, Restom, Liau, & Liu, 2007) to eliminate the nonneuronal contributions from WM and CSF, followed by band‐pass filtering (0.008 < *f* < 0.09 Hz). Denoising also included the regression of time points flagged as outliers due to motion, along with the seven realignment parameters (three translations, three rotations, and one composite motion) and their first‐order derivatives. In‐house custom software ART version 2015‐10 (Artifact Detection Tools (ART) (n.d.)) was used for outlier detection, with thresholds defined using the 99th percentile settings, and allowed for the quantification of participant motion in the scanner and the identification of outliers based on subject motion as well as changes in the mean blood oxygen level‐dependent (BOLD) signal. With these settings, outlier scans were identified as consecutive scans with global‐signal changes above 9 *SD*s or framewise displacement above 2 mm. Subjects were excluded if they had >20% of movement outliers, thus falling short of the minimum required scan time length for resting state connectivity analysis (Airan et al., 2016; Van Dijk et al., 2010). Motion information and framewise outliers were included as nuisance covariates in our subsequent first‐level analyses. After denoising, the residual BOLD time courses from the networks were extracted to obtain correlation maps.

#### 2.11.2 Functional connectivity analyses

**FIGURE 2:**
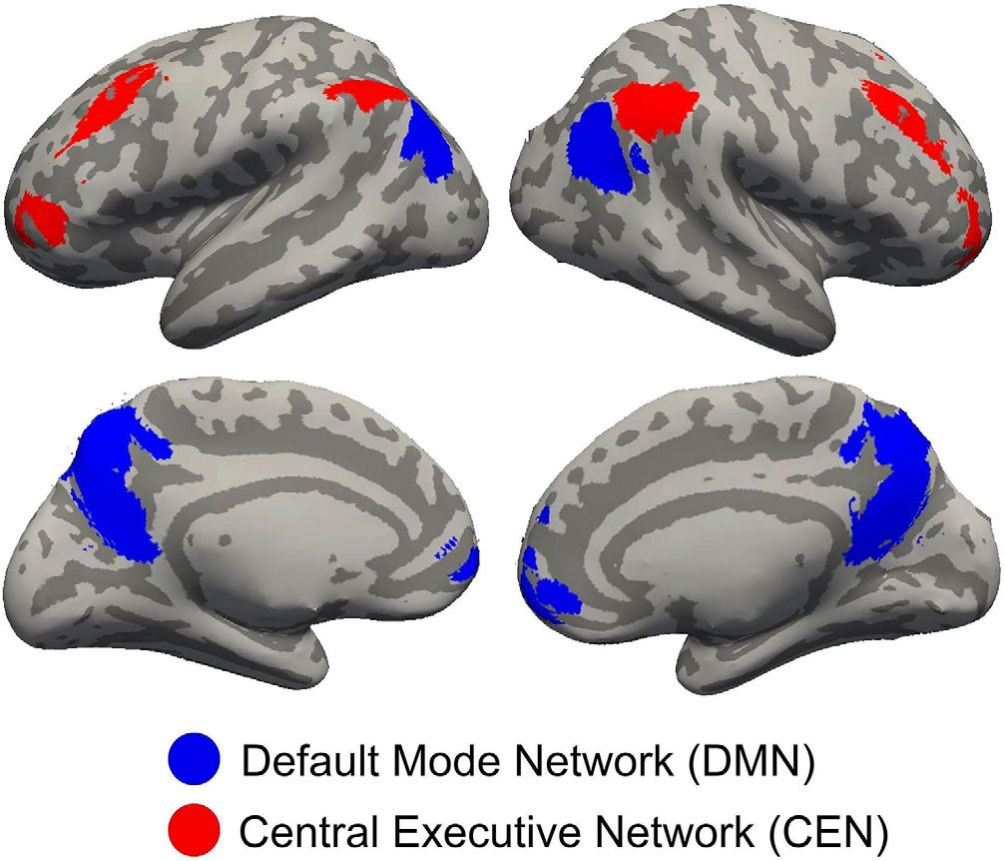
Left and right lateral and medial inflated views of the brain showing default mode network (DMN) (blue clusters) and central executive network (red) nodes used as regions of interest (ROIs) derived from the Human Connectome Project that were used to assess changes in DMN–CEN anticorrelation

Functional connectivity analysis was performed using CONN 18.b (Whitfield‐Gabrieli & Nieto‐Castanon, 2012) toolbox. We examined functional connectivity between *a prior* regions of interest (ROIs or nodes) in the DMN and CEN networks. Node regions for both networks were defined by the CONN software package and derived from an independent component analysis on 497 healthy control participants (293 females) as part of the Human Connectome Project (http://www.humanconnectome.org). DMN nodes (blue clusters in Figure 2) included MPFC, PCC, left and right parietal cortex (LPC; RPC). These four DMN nodes were combined into a single seed for analysis. The four CEN nodes as ROI's (red clusters in Figure 2), which included left and right prefrontal cortices (LPFC; RPFC) and left and right posterior parietal cortices (LPPC; RPPC), were also combined into a single mask for further analyses using small volume correction (SVC).

For first‐level functional connectivity analysis, Pearson's correlation coefficients were generated by computing correlations between the DMN time series and time series of all other voxels in the brain. These seed‐to‐voxel *r* maps were then transformed to *z* maps using Fisher's *r*‐to‐*z* transformation and brought up to a general linear model analysis at the second level for within‐group and between‐group comparisons. Finally, we performed an SVC (Poldrack, 2007; Worsley et al., 1996) on the a priori defined CEN mask.

#### 2.11.3 Pre‐intervention relation of Go‐Accuracy to DMN connectivity

To investigate the relationship between pre‐intervention Go‐Accuracy and DMN connectivity, we correlated the pre‐intervention Go‐Accuracy with the random effects connectivity maps at pre‐intervention from the DMN network from all participants.

#### 2.11.4 Effect of training on DMN–CEN anticorrelation

Regression analysis was used to assess the causal impact of mindfulness training. The model regressed connectivity maps from the DMN network on intervention group assignment (1 for mindfulness training, 0 for coding training). Significant main effects and interactions were followed up with post hoc testing. All analyses controlled for pre‐intervention performance, gender, and IQ to determine beta coefficients of the treatment effect. Unless otherwise stated all statistical analysis are nonparametric (1,000 permutations) with a height threshold of *p* < .05 at the voxel level and an extent threshold of FWE‐corrected *p* < .05.

## 3 RESULTS

### 3.1 Student acceptability of interventions

Both groups reported similar acceptability for the mindfulness or coding training. There were no significant differences for the overall rating of the class (*t*(25) = 1.22, *p* = .23; mean and *SD* values for mindfulness group: 3.23 (1.23) and coding group: 2.7 (1.49)), the amount of work they had to do (*t*(25) = 1.60, *p* = .12; mindfulness group: 2.6 (1.12) and coding group: 3.3 (0.82)), degree of active participation (*t*(25) = .75, *p* = .45; mindfulness group: 3.1 (1.28) and coding group: 3.8 (.78)), and how much practical knowledge they learned (*t*(25) = 1.85, *p* = .08; mindfulness group: 2.6 (1.31) and coding group: 3.6 (.69)). These results show that there were no significant differences in student perceptions of the two training courses.

### 3.2 Effect of mindfulness training program on SART performance

#### 3.2.1 Go‐Accuracy

**FIGURE 3:**
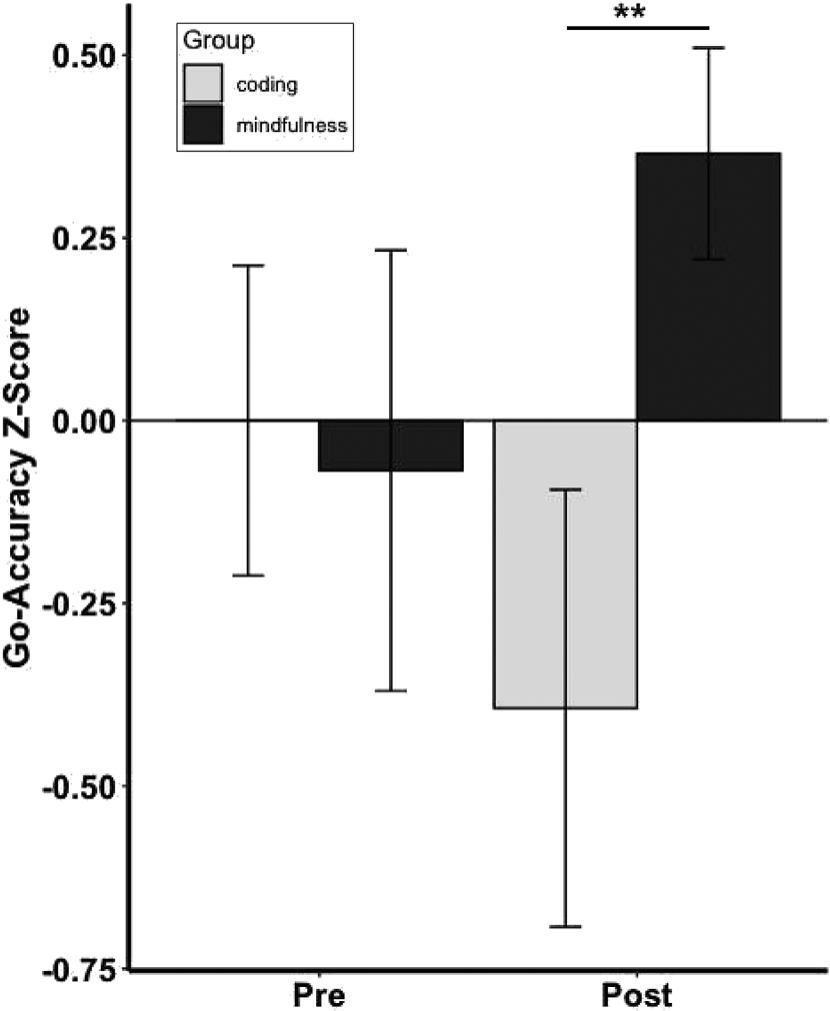
Pre‐intervention (pre) and post‐intervention (post) Z‐scores on Go‐Accuracy for the Sustained Attention to Response Task (SART) for mindfulness‐training and coding‐training groups. Statistics are linear regressions taking into account pre‐intervention performance as covariate as well as IQ and gender. Error bars represent *SE*. **p < .01

At baseline, Go‐Accuracy was significantly less than 100% (*t*(30) = −7.45, *p* = 2.63e‐08, Cohen *d =* 1.89). The mindfulness‐training group exhibited significantly better Go‐Accuracy after the intervention compared to the coding‐training group (*b* = .89, *t*(25) = 2.57, *p* = .01, Cohen *f* ^2^ *=* .47; Figure 3). This was confirmed by post hoc two‐sample *t* tests between the groups showing no significant difference at pre‐intervention for Go‐Accuracy (*t*(30) = .21, *p* = .83), but significantly better Go‐Accuracy at post‐intervention (*t*(30) = 2.28, *p* = .01) in the mindfulness‐training group than the coding‐training group. Additionally, post hoc paired *t* tests revealed that children who received the mindfulness‐training intervention did not exhibit any significant pre–post intervention decline in Go‐Accuracy (*t*(14) = 1.50, *p* = .92), whereas children in the coding‐training group exhibited a significant pre–post decline in Go‐Accuracy (*t*(15) = 1.87, *p* = .04). There were no group differences in RT variability on Go trials both before the intervention (*t*(30) = .09, *p* = .92) or after the intervention (*b* = .02, *t*(25) = .97, *p* = .33, Cohen *f* ^2^ *=* .01).

#### 3.2.2 No‐Go‐Accuracy

There were no significant group differences in No‐Go‐Accuracy before (*t*(30) = .91, *p* = .36) or after the intervention (*b* = .05, *t*(25) = .12, *p* = .90, Cohen *f* ^2^ *=* .01) and on No‐Go‐RT‐variability before (*t*(30) = .05, *p* = .95) or after the intervention (*b* = .24, *t*(25) = 1.53, *p* = .13, Cohen *f* ^2^ *=* .17).

### 3.3 Functional connectivity analysis

#### 3.3.1 Motion

There were seven participants who exceeded the 20% movement threshold of images (or less than 132 usable time points) with movement outliers (4 mindfulness) and were discarded from the analysis. No significant effects of time (pre‐intervention vs. post‐intervention; *F*(1,25) = .72; *p* = .48), group (mindfulness versus coding; *F*(1,25) = .98; *p* = .33), and no significant interaction effects (*F*(1,25) = .84; *p* = .40) were observed (median value mindfulness group at pre‐intervention: 6.0, at post 6.0; coding group at pre‐intervention: 6.2, at post 7.8). These results show that there were no significant group differences in motion. There was also no correlation between Motion and Go‐Accuracy (*r* = .1, *p* = .5).

#### 3.3.2 Pre‐intervention relation of SART performance to DMN–CEN anticorrelation

**FIGURE 4:**
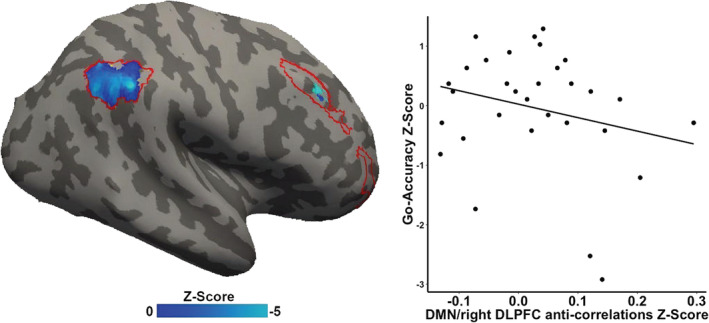
Relation of Sustained Attention to Reaction Task (SART) performance on Go‐Accuracy to default mode network (DMN) and central executive network (CEN red outline) anticorrelation. Inflated right hemisphere of the brain depicting voxels in the right dorsolateral prefrontal cortex (right DLPFC) and right parietal cortex (blue) in which greater baseline anticorrelation correlated significantly with better SART Go‐Accuracy. Statistics are nonparametric FWE small volume corrected

Across participants, prior to intervention, better Go‐Accuracy correlated with greater anticorrelation between DMN–CEN (*r* = −.45, *n* = 31, *p* = .005, FWE‐corrected; Figure 4; specifically in right DLPFC (MNI x = 52, y = 26, z = 36) and rPPC (MNI x = 56, y = −50, z = 44). There were no significant correlations between DMN–CEN functional connectivities and No‐Go‐accuracy (*r* = .33, *n* = 31, *p* = .96, FWE‐corrected).

#### 3.3.3 Effect of mindfulness training on DMN–CEN anticorrelation

**FIGURE 5:**
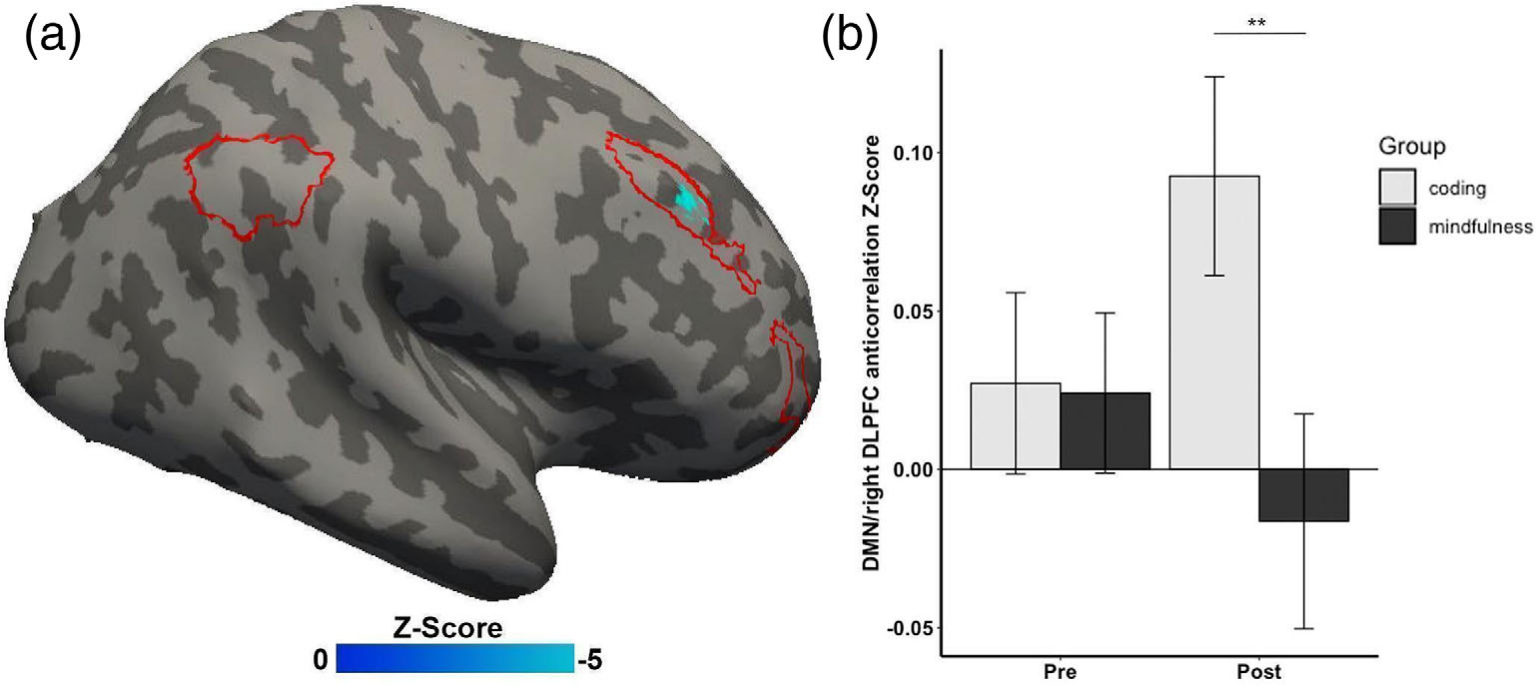
Regions exhibiting significant differences of default mode network (DMN) and central executive network (CEN) anticorrelation between the mindfulness training group versus the coding training group. (a) Inflated right hemisphere of the brain depicting voxels in the right dorsolateral prefrontal cortex (right DLPFC in blue) within the CEN (red outline) where DMN–CEN anticorrelation showed a significant group difference. (b) Significant difference in pre‐to‐post‐intervention DMN–CEN anticorrelation in the mindfulness training group relative to the coding training group. Statistics are nonparametric FWE small volume corrected. All centers reflect mean and all error bars reflect the *SEM*. **p < .01

The mindfulness‐training group showed a significant pre–post increase in DMN–CEN anticorrelation (right DLPFC: x = 50, y = 22, z = 35) after the intervention compared to the coding‐training group (*b* = −.14, *t*(25) = −3.35, *p* = .002, Cohen *f* ^2^ *=* .56, FWE‐corrected; Figure 5). This was confirmed by post hoc two‐sample *t* tests between the groups showing no significant difference at pre‐intervention in DMN‐right DLPFC anticorrelation (*t*(30) = .07, *p* = .93), but significantly higher DMN‐right DLPFC anticorrelation at post‐intervention (*t*(30) = 2.35, *p* = .02) in the mindfulness‐training group than the coding‐training group. Additionally, post hoc paired *t* tests revealed that children in the mindfulness‐training group did not exhibit any significant post‐intervention decline in DMN‐right DLPFC anticorrelation (*t*(14) = .92, *p* = .81), whereas children in the coding‐training group exhibited a significant reduction in post‐intervention DMN–CEN anticorrelation (*t*(15) = 1.87, *p* = .04).

Given the importance of motion as a possible confound in functional connectivity, we performed analyses following Ciric et al. (2017) to evaluate the efficacy of our denoising strategy (see Figures S1.1 and S2.2, Supplementary Material). Additionally, we performed a post hoc analysis using the subject‐level motion covariate as a control variable to evaluate whether results on DMN‐right DLPFC anticorrelations were affected by differences in motion among participants. The finding of a group × pre–post interaction remained significant (*b* = −.15, *t*(24) = −3.69, *p* = .001, Cohen *f* ^2^ = .59, FWE‐corrected). Furthermore, there was no significant difference between results with or without motion covariate as control variable (*delta* = .01, *SE* = .05, *Z* = .20, *p* = .83).

#### 3.3.4 Relation between change in Go‐Accuracy accuracy to change in DMN‐right DLPFC anticorrelation

**FIGURE 6:**
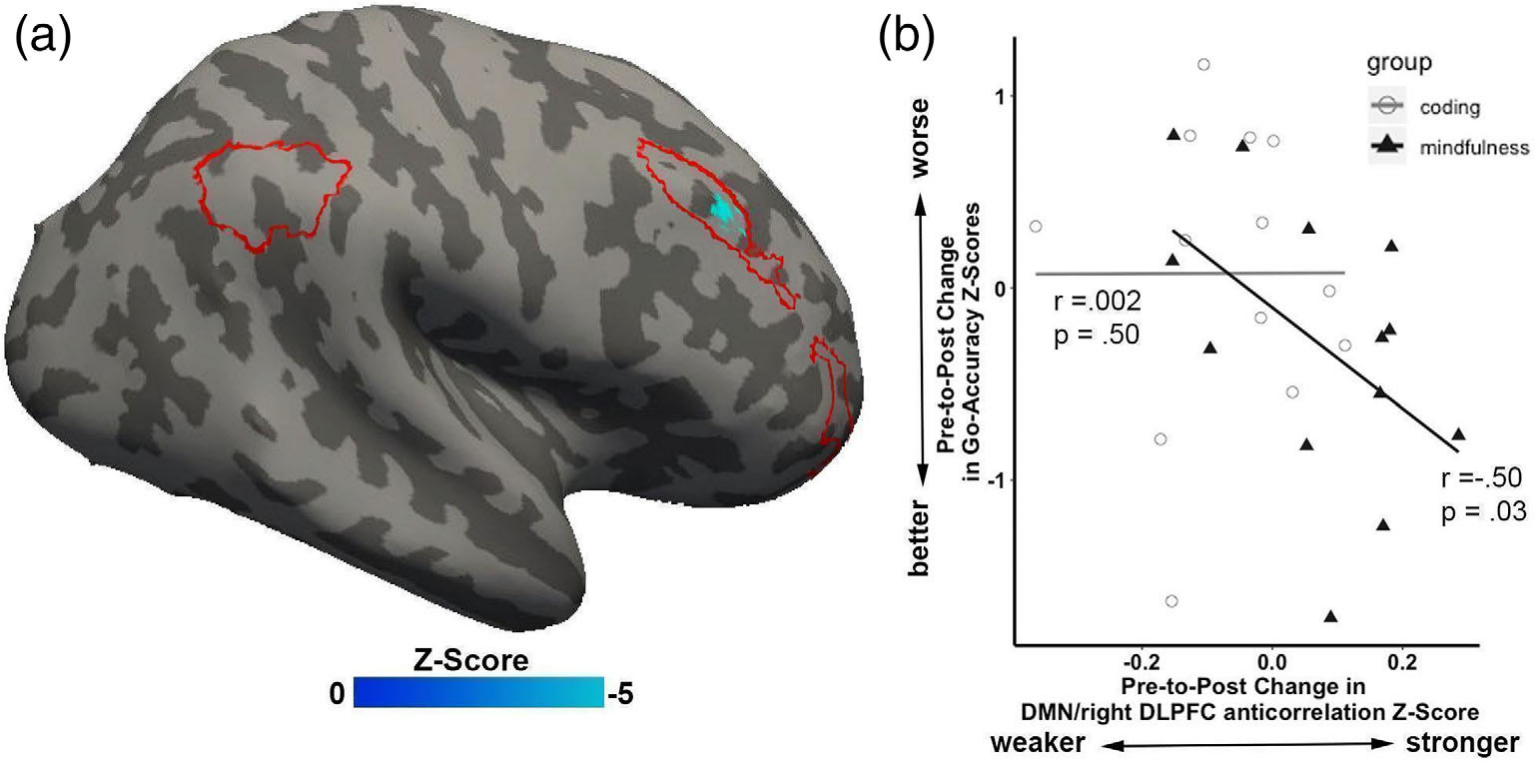
Pre–post changes in Sustained Attention to Response Task (SART) Go‐Accuracy and in default mode network (DMN) and right dorsolateral prefrontal cortex (DLPFC) anticorrelation for the mindfulness (filled triangles) and coding (open circles) training groups. Only children in the mindfulness group exhibited a significant correlation between changes in pre–post SART performance and changes in pre–post anticorrelation between DMN and right DLPFC

Only children in the mindfulness‐training group showed a significant and positive correlation between pre–post differences in Go‐Accuracy and pre–post differences in DMN‐right DLPFC anticorrelation (right DLPFC: x = 50, y = 22, z = 35, mindfulness training: *r* = −.50, *p* = .03; coding training: *r* = .002, *p* = .50; Figure 6).

## 4 DISCUSSION

We discovered a neural network characteristic associated with variation in sustained attention in children, and through an RCT design found novel causal evidence that mindfulness training, relative to coding training, preserved sustained attention in association with preservation of that neural network characteristic. There were three major findings. First, prior to intervention, better sustained attention positively correlated with greater resting‐state anticorrelation between two distinct brain networks across all children: the DMN (associated with mind‐wandering and task‐unrelated thoughts) and the right DLPFC and right parietal components of the CEN (associated with cognitive control). This is the first evidence linking performance on an attention task with rsFC in children. Second, children who participated in an 8‐week long mindfulness training exhibited preserved sustained attention, whereas children in the active control condition exhibited a deterioration of sustained attention. Third, and mirroring the preservation of sustained attention, children who received mindfulness training exhibited preserved resting‐state anticorrelation between the DMN and right DLPFC, whereas children in the active control condition exhibited a deterioration of that anticorrelation. The relations among mindfulness training, sustained attention, and brain plasticity received further support by a correlation between changes in sustained attention performance and changes in DMN‐right DLPFC anticorrelation only in the children who received mindfulness training. Altogether, this study provides initial causal evidence for the brain basis of cognitive benefits due to mindfulness training in children.

### 4.1 Greater sustained attention correlated with greater resting‐state DMN‐right DLPFC anticorrelation prior to intervention

The present findings provide initial evidence about how variation in sustained attention among children relates to variation in brain function. Prior to intervention and across all children, greater sustained attention was associated with greater resting‐state anticorrelation between the DMN and a major hub of the CEN, right DLPFC. This brain–behavior relation is consistent with prior findings in adults that the DMN and CEN play key roles in attentional processes and in individual differences in cognitive control. DMN–DLPFC anticorrelation is enhanced during attention‐demanding tasks (Denkova et al., 2019; Greicius et al., 2003; Piccoli et al., 2015), and across individual adults stronger resting‐state DMN–DLPFC anticorrelation is associated with greater working memory capacity (Hampson et al., 2010; Keller et al., 2015; Whitfield‐Gabrieli et al., 2018) and faster processing speed (Ng et al., 2016). Conversely, such DMN–DLPFC anticorrelation is reduced when cognitive control processes are clinically impaired in individuals with ADHD (Hoekzema et al., 2014; Mattfeld et al., 2014) or schizophrenia (Whitfield‐Gabrieli et al., 2009).

The relation of DMN–CEN anticorrelation to sustained attention may be interpreted through the development of this anticorrelation in children and adolescents. One study compared resting‐state anticorrelation between a hub of the DMN (MPFC) and multiple CEN regions in children ages 8–12, adolescents ages 13–17, and young adults ages 18–24 (Chai, Ofen, Gabrieli, & Whitfield‐Gabrieli, 2014). In children, these areas were positively correlated, and adolescents exhibited an intermediate level of anticorrelation relative to the anticorrelation seen in adults. A longitudinal study examining changes in functional connectivity across ages 10–13 while children performed a passive listing tasks reported a similar growth of anticorrelation between PCC, another hub of the DMN, and CEN regions that correlated with IQ scores (Sherman et al., 2014). These studies converge to suggest that DMN–CEN relations mature through development from a positive correlation to a negative correlation, and that this maturation of DMN–CEN anticorrelation is associated with the growth of cognitive control.

### 4.2 Mindfulness training preserved sustained attention

Mindfulness training preserved sustained attention on the SART in the mindfulness group relative to the coding group. This finding aligns with prior behavioral studies reporting enhanced cognitive control after mindfulness training in children (Britton et al., 2014; Felver et al., 2017; Flook et al., 2010; Lawler et al., 2019; Leonard et al., 2013; Quach et al., 2016; Salmoirago‐Blotcher et al., 2019; Schonert‐Reichl et al., 2015;Semple et al., 2010; Sidhu, 2012) and adults (Cásedas et al., 2019; Moore & Malinowski, 2009). These RCTs demonstrated improved attentional ability measured by task performance or parent/teacher‐reported questionnaires in typically developing children and adolescents (Felver et al., 2017; Schonert‐Reichl et al., 2015), in children with ADHD (Sidhu, 2012) or reading difficulties (Semple et al., 2010; Sidhu, 2012), and in incarcerated adolescents (Leonard et al., 2013).

In the current study, behavioral performance followed a particular pattern, with the two groups performing similarly at pre‐intervention. At post‐intervention, the mindfulness group maintained similar levels of sustained attention, whereas sustained attention declined in the active control group. One interpretation is that both groups, during the 15 min of the arduous and attention‐demanding performance at pre‐intervention, came to realize the difficulty of the task. This realization may have subsequently diminished engagement in the task during the post‐intervention readministration of the SART. Thus, simply maintaining a similar level of sustained attention in the second SART testing indicates achievement instead of a lack of improvement. Indeed, other studies with adults involving pre–post SART designs have also reported significant worsening of performance in control groups across sessions with mindfulness resulting in significant improvement by virtue of maintaining pre‐intervention levels of sustained attention (Hargus, Crane, Barnhofer, & Williams, 2010; Jha et al., 2015; Polak, 2009).

Deterioration of sustained attention in the control group may also be related to contextual factors of the academic calendar. Pre‐intervention measures were obtained from December through January, and post‐intervention measures were obtained from April through May. Broadly, the post‐intervention period overlapped with the most stressful part of the school year as students take statewide tests of academic achievement and prepare for the transition to the next school year. It may be that a rising general level of stress for all students across the time period of this study accounted for the worsening of sustained attention in the control group. These observations suggest that mindfulness may help buffer increases in stress and negative affect that occur across the academic year, and that mindfulness training was a protective factor for students.

### 4.3 Mindfulness training preserved DMN‐right DLPFC anticorrelation

Group‐differences in the strength of resting‐state DMN‐right DLPFC anticorrelation paralleled the behavioral findings for sustained attention. The mindfulness group maintained pre‐intervention levels of DMN‐right DLPFC anticorrelation, whereas the coding group exhibited reduced anticorrelations. This link between mindfulness training and intervention‐related changes in brain–behavior associations was further supported by a correlation between changes in SART performance and changes in DMN‐right DLPFC correlation that occurred only in the mindfulness group. This finding, to our knowledge, is the first evidence of a causal relationship between changes in sustained attention and changes in DMN‐right DLPFC anticorrelations, in any age group. Previous studies reported a causal link between greater DMN activations and poorer vigilance (Hinds et al., 2013), and showed a causal inhibitory regulation of the CEN on DMN activations and connectivity patterns (Chen et al., 2013). Another study found increased DMN‐right DLPFC anticorrelation in patients with schizophrenia following an intervention of cannabis consumption (Whitfield‐Gabrieli et al., 2018). This study found that the anticorrelation correlated with working‐memory performance after intervention, but changes in performance were not correlated with changes in functional connectivity. Several prior studies have reported changes in rsFC in adults following mindfulness training, but the absence of behavioral measures precluded relating those changes in rsFC to any cognitive functions (Creswell et al., 2016; Taren et al., 2015; Taren et al., 2017; Yang et al., 2016). Given that the correlation between DMN–CEN transitions from positive to negative across early adolescent brain development (Chai et al., 2014; Sherman et al., 2014), our findings raise the possibility that mindfulness promotes the maturation of the neural circuits associated with cognitive control.

It is unclear why the coding group exhibited a decline in the DMN‐right DLPFC anticorrelation following intervention. It is unlikely that the coding training itself diminished the anticorrelation because students rated the demands and enjoyment of the two interventions very similarly. Also, a lack of correlation between changes in SART performance and changes in anticorrelation in the coding group suggests that there was not a systematic relation between coding training and either behavioral or brain changes. One possibility is that the greater level of stress in the school year not only diminished sustained attention, but also the brain network connectivity (i.e., DMN‐right DLPFC anticorrelation) that supports sustained attention. In this case, mindfulness training may be seen as a protective factor against such neurocognitive effects of stress, as it is for behavioral effects of stress (Jha, Stanley, Kiyonaga, Wong, & Gelfand, 2010).

### 4.4 Limitations and implications

Several limitations of this study can be noted. First, there were a modest number of participants. In turn, this motivated an a priori approach to generate specific hypotheses about the neural networks that may change following a mindfulness intervention, so as to allow for a conservative level of statistics. It is unknown, therefore, whether other neural networks would also display training‐induced resting‐state plasticity. In addition, we utilized a network approach whereby all four seeds of the same network were analyzed together, in order to minimize the number of comparisons to be conducted. Second, although the study had an RCT design, the findings are from the subset of families who were willing to participate in neuroimaging. This resulted in an imbalance (although nonsignificant) in gender ratios between the two groups. However, we mitigated the effect of this imbalance by adding gender as a covariate in all analyses. Any additional characteristics that may have distinguished these families were equivalent across the two training groups.

The study also has several strengths. First, it generalizes the benefits from mindfulness training beyond both active engagement in meditation and task‐specific brain plasticity. The changes in behavior and brain function occurred in a nonmeditative state. These findings are in agreement with the notion that mindfulness training transfers its effects to daily experiences beyond meditation practice (Lutz, Brefczynski‐Lewis, Johnstone, & Davidson, 2008; Lutz, Dunne, & Davidson, 2007). Further, the observed functional brain differences were not limited to a specific task or activity because rsFC is thought to reflect primarily long‐term, tonic network properties of neural systems that are shaped by experience and development and that have broad consequences for behaviors (Chai et al., 2014; Sherman et al., 2014). A second strength of this study is the association between a behavioral measure of attention and a separate measure of brain function. The finding of an objective neural correlate of sustained attention which tracks the beneficial behavioral effects following mindfulness intervention supports the validity of the behavioral results at both group and individual differences levels of analysis. Finally, given that mindfulness training appears to have conferred a protective effect on sustained attention and DMN‐right DLPFC anticorrelation, this finding emphasizes the value of including a randomized control group that helped to establish a true baseline against which a treatment effect could be discerned.

The present study found that a grade‐wide, school‐based mindfulness program preserved cognitive performance, which has important implications for mental health and educational practices. This is further corroborated by a previously reported finding on the impact of this intervention on social–emotional outcomes of reduced stress and reduced negative affect (Bauer et al., 2019). Indeed, the interaction between cognitive control and social–emotional functions are important in adolescent development. Reduced cognitive control in emotional contexts in adolescence has been associated with risk‐taking behaviors, mental disorders, mortality, and crime (Coleman, 2011; Paus, Keshavan, & Giedd, 2008; Rudolph et al., 2017), whereas greater cognitive control has been linked to academic and professional success (Caspi, Entner Wright, Moffitt, & Silva, 1998; Finn et al., 2014; Finn et al., 2017; Moffitt et al., 2011). Finally, this RCT occurred at an urban school serving many students from lower income (low socioeconomic status) families, which was also reflected in the subgroup of students who participated in the imaging study. Thus, mindfulness training may be especially helpful in supporting cognitive control in students who may experience higher rates of early‐life adversity. The present findings point to the neural mechanisms of how mindfulness training may promote healthy development of cognitive control as well as enhance well‐being and academic achievement in youth.

## CONFLICT OF INTEREST

The authors declare no conflict of interest.

## Supporting information


**Figure S1.1** Residual correlation across subjects between Functional Connectivity (FC) and a Quality Control measure (QC) indicative of average subject motion, before (top) and after (bottom) denoising of the BOLD signal.
**Figure S1.2**. Distributions of functional connectivity values (bivariate correlation coefficients) in a network of 512 nodes across the entire brain, computed separately for each subject during pre‐ (top) and post‐ (bottom) sessions.

## Data Availability

The data that support the findings of this study are available from the corresponding author upon reasonable request.

## References

[hbm25197-bib-0001] Airan, R. D. , Vogelstein, J. T. , Pillai, J. J. , Caffo, B. , Pekar, J. J. , & Sair, H. I. (2016). Factors affecting characterization and localization of interindividual differences in functional connectivity using MRI. Human Brain Mapping, 37, 1986–1997. 27012314 10.1002/hbm.23150 PMC5516891

[hbm25197-bib-0002] Allan Cheyne, J. , Solman, G. J. F. , Carriere, J. S. A. , & Smilek, D. (2009). Anatomy of an error: A bidirectional state model of task engagement/disengagement and attention‐related errors. Cognition, 111, 98–113. 19215913 10.1016/j.cognition.2008.12.009

[hbm25197-bib-0003] Artifact Detection Tools (ART) . (n.d.). Retrieved from https://www.nitrc.org/projects/artifact_detect/.

[hbm25197-bib-0004] Ashburner, J. , & Friston, K. J. (2005). Unified segmentation. NeuroImage, 26, 839–851. 15955494 10.1016/j.neuroimage.2005.02.018

[hbm25197-bib-0005] Bastian, M. , & Sackur, J. (2013). Mind wandering at the fingertips: Automatic parsing of subjective states based on response time variability. Frontiers in Psychology, 4, 573. 24046753 10.3389/fpsyg.2013.00573 PMC3763218

[hbm25197-bib-0006] Bauer, C. C. C. , Caballero, C. , Scherer, E. , West, M. R. , Mrazek, M. D. , Phillips, D. T. , … Gabrieli, J. D. E. (2019). Mindfulness training reduces stress and amygdala reactivity to fearful faces in middle‐school children. Behavioral Neuroscience, 133, 569–585. 31448928 10.1037/bne0000337

[hbm25197-bib-0007] Behzadi, Y. , Restom, K. , Liau, J. , & Liu, T. T. (2007). A component based noise correction method (CompCor) for BOLD and perfusion based fMRI. NeuroImage, 37, 90–101. 17560126 10.1016/j.neuroimage.2007.04.042 PMC2214855

[hbm25197-bib-0008] Bluth, K. , Campo, R. A. , Pruteanu‐Malinici, S. , Reams, A. , Mullarkey, M. , & Broderick, P. C. (2016). A school‐based mindfulness pilot study for ethnically diverse at‐risk adolescents. Mindfulness, 7, 90–104. 27034729 10.1007/s12671-014-0376-1 PMC4809539

[hbm25197-bib-0009] Britton, W. B. , Lepp, N. E. , Niles, H. F. , Rocha, T. , Fisher, N. E. , & Gold, J. S. (2014). A randomized controlled pilot trial of classroom‐based mindfulness meditation compared to an active control condition in sixth‐grade children. Journal of School Psychology, 52, 263–278. 24930819 10.1016/j.jsp.2014.03.002 PMC4060047

[hbm25197-bib-0010] Calmer Choice (n.d.). Retrieved from http://www.calmerchoice.org.

[hbm25197-bib-0011] Cásedas, L. , Pirruccio, V. , Vadillo, M. A. , & Lupiáñez, J. (2019). Does mindfulness meditation training enhance executive control? A systematic review and meta‐analysis of randomized controlled trials in adults. Mindfulness, 11, 411–424. 10.1007/s12671-019-01279-4

[hbm25197-bib-0012] Caspi, A. , Entner Wright, B. R. , Moffitt, T. E. , & Silva, P. A. (1998). Early failure in the labor market: Childhood and adolescent predictors of unemployment in the transition to adulthood. American Sociological Review, 63, 424.

[hbm25197-bib-0013] Chai, X. J. , Ofen, N. , Gabrieli, J. D. E. , & Whitfield‐Gabrieli, S. (2014). Selective development of anticorrelated networks in the intrinsic functional organization of the human brain. Journal of Cognitive Neuroscience, 26, 501–513. 24188367 10.1162/jocn_a_00517 PMC4175987

[hbm25197-bib-0014] Chao, L. L. , & Knight, R. T. (1998). Contribution of human prefrontal cortex to delay performance. Journal of Cognitive Neuroscience, 10, 167–177. 9555105 10.1162/089892998562636

[hbm25197-bib-0015] Chen, A. C. , Oathes, D. J. , Chang, C. , Bradley, T. , Zhou, Z.‐W. , Williams, L. M. , … Etkin, A. (2013). Causal interactions between fronto‐parietal central executive and default‐mode networks in humans. Proceedings of the National Academy of Sciences of the United States of America, 110, 19944–19949. 24248372 10.1073/pnas.1311772110 PMC3856839

[hbm25197-bib-0016] Cheyne, J. A. , Carriere, J. S. A. , Solman, G. J. F. , & Smilek, D. (2011). Challenge and error: Critical events and attention‐related errors. Cognition, 121, 437–446. 21861999 10.1016/j.cognition.2011.07.010

[hbm25197-bib-0017] Chiesa, A. , Calati, R. , & Serretti, A. (2011). Does mindfulness training improve cognitive abilities? A systematic review of neuropsychological findings. Clinical Psychology Review, 31, 449–464. 21183265 10.1016/j.cpr.2010.11.003

[hbm25197-bib-0018] Christoff, K. , Gordon, A. M. , Smallwood, J. , Smith, R. , & Schooler, J. W. (2009). Experience sampling during fMRI reveals default network and executive system contributions to mind wandering. Proceedings of the National Academy of Sciences of the United States of America, 106, 8719–8724. 19433790 10.1073/pnas.0900234106 PMC2689035

[hbm25197-bib-0019] Ciric, R. , Wolf, D. H. , Power, J. D. , Roalf, D. R. , Baum, G. L. , Ruparel, K. , … Satterthwaite, T. D. (2017). Benchmarking of participant‐level confound regression strategies for the control of motion artifact in studies of functional connectivity. NeuroImage, 154, 174–187. 28302591 10.1016/j.neuroimage.2017.03.020 PMC5483393

[hbm25197-bib-0020] Coleman, J. C. (2011). The nature of adolescence (4th ed). Abingdon‐on‐Thames, England: Taylor & Francis.

[hbm25197-bib-0021] Creswell, J. D. , Taren, A. A. , Lindsay, E. K. , Greco, C. M. , Gianaros, P. J. , Fairgrieve, A. , … Ferris, J. L. (2016). Alterations in resting‐state functional connectivity link mindfulness meditation with reduced interleukin‐6: A randomized controlled trial. Biological Psychiatry, 80, 53–61. 27021514 10.1016/j.biopsych.2016.01.008

[hbm25197-bib-0022] Denkova, E. , Nomi, J. S. , Uddin, L. Q. , & Jha, A. P. (2019). Dynamic brain network configurations during rest and an attention task with frequent occurrence of mind wandering. Human Brain Mapping, 40, 4564–4576. 31379120 10.1002/hbm.24721 PMC6865814

[hbm25197-bib-0023] Duncan, G. J. , Dowsett, C. J. , Claessens, A. , Magnuson, K. , Huston, A. C. , Klebanov, P. , … Japel, C. (2008). “School readiness and later achievement”: Correction to Duncan et al. (2007). Developmental Psychology, 44, 232–232. 10.1037/0012-1649.43.6.1428 18020822

[hbm25197-bib-0024] Dweck, C. S. (2006). Mindset: The new psychology of success, New York, NY: Random House.

[hbm25197-bib-0025] Felver, J. C. , Tipsord, J. M. , Morris, M. J. , Racer, K. H. , & Dishion, T. J. (2017). The effects of mindfulness‐based intervention on children's attention regulation. Journal of Attention Disorders, 21, 872–881. 25172884 10.1177/1087054714548032

[hbm25197-bib-0026] Finn, A. S. , Kraft, M. A. , West, M. R. , Leonard, J. A. , Bish, C. E. , Martin, R. E. , … Gabrieli, J. D. E. (2014). Cognitive skills, student achievement tests, and schools. Psychological Science, 25, 736–744. 24434238 10.1177/0956797613516008 PMC3954910

[hbm25197-bib-0027] Finn, A. S. , Minas, J. E. , Leonard, J. A. , Mackey, A. P. , Salvatore, J. , Goetz, C. , … Gabrieli, J. D. E. (2017). Functional brain organization of working memory in adolescents varies in relation to family income and academic achievement. Developmental Science, 20, e12450. 10.1111/desc.12450 27434857

[hbm25197-bib-0028] Finucane, A. , & Mercer, S. W. (2006). An exploratory mixed methods study of the acceptability and effectiveness of mindfulness‐based cognitive therapy for patients with active depression and anxiety in primary care. BMC Psychiatry, 6, 14. 16603060 10.1186/1471-244X-6-14 PMC1456957

[hbm25197-bib-0029] Flook, L. , Smalley, S. L. , Jennifer Kitil, M. , Galla, B. M. , Kaiser‐Greenland, S. , Locke, J. , … Kasari, C. (2010). Effects of mindful awareness practices on executive functions in elementary school children. Journal of Applied School Psychology, 26, 70–95.

[hbm25197-bib-0030] Fox, M. D. , & Raichle, M. E. (2007). Spontaneous fluctuations in brain activity observed with functional magnetic resonance imaging. Nature Reviews. Neuroscience, 8, 700–711. 17704812 10.1038/nrn2201

[hbm25197-bib-0031] Fox, M. D. , Snyder, A. Z. , Vincent, J. L. , Corbetta, M. , van Essen, D. C. , & Raichle, M. E. (2005). From the cover: The human brain is intrinsically organized into dynamic, anticorrelated functional networks. Proceedings of the National Academy of Sciences of the United States of America, 102, 9673–9678. 15976020 10.1073/pnas.0504136102 PMC1157105

[hbm25197-bib-0032] Friston, K. (2007). A short history of SPM In Statistical parametric mapping (pp. 3–9). Boston, MA: Elsevier / Academic Press.

[hbm25197-bib-0033] Geier, C. F. , Garver, K. , Terwilliger, R. , & Luna, B. (2009). Development of working memory maintenance. Journal of Neurophysiology, 101, 84–99. 18971297 10.1152/jn.90562.2008 PMC2637004

[hbm25197-bib-0034] Giannopulu, I. , Escolano, S. , Cusin, F. , Citeau, H. , & Dellatolas, G. (2008). Teachers' reporting of behavioural problems and cognitive‐academic performances in children aged 5‐7 years. British Journal of Educational Psychology, 78, 127–147. 17535517 10.1348/000709907X204372

[hbm25197-bib-0035] Greicius, M. D. , Krasnow, B. , Reiss, A. L. , & Menon, V. (2003). Functional connectivity in the resting brain: A network analysis of the default mode hypothesis. Proceedings of the National Academy of Sciences of the United States of America, 100, 253–258. 12506194 10.1073/pnas.0135058100 PMC140943

[hbm25197-bib-0036] Hampson, M. , Driesen, N. , Roth, J. K. , Gore, J. C. , & Constable, R. T. (2010). Functional connectivity between task‐positive and task‐negative brain areas and its relation to working memory performance. Magnetic Resonance Imaging, 28, 1051–1057. 20409665 10.1016/j.mri.2010.03.021 PMC2936669

[hbm25197-bib-0037] Hargus, E. , Crane, C. , Barnhofer, T. , & Williams, J. M. G. (2010). Effects of mindfulness on meta‐awareness and specificity of describing prodromal symptoms in suicidal depression. Emotion, 10, 34–42. 20141300 10.1037/a0016825 PMC3933215

[hbm25197-bib-0038] Hinds, O. , Thompson, T. W. , Ghosh, S. , Yoo, J. J. , Whitfield‐Gabrieli, S. , Triantafyllou, C. , & Gabrieli, J. D. E. (2013). Roles of default‐mode network and supplementary motor area in human vigilance performance: Evidence from real‐time fMRI. Journal of Neurophysiology, 109, 1250–1258. 23236006 10.1152/jn.00533.2011

[hbm25197-bib-0039] Hoekzema, E. , Carmona, S. , Antoni Ramos‐Quiroga, J. , Fernández, V. R. , Bosch, R. , Soliva, J. C. , … Vilarroya, O. (2014). An independent components and functional connectivity analysis of resting state fMRI data points to neural network dysregulation in adult ADHD. Human Brain Mapping, 35, 1261–1272. 23417778 10.1002/hbm.22250 PMC6869838

[hbm25197-bib-0040] Jha, A. P. , Morrison, A. B. , Dainer‐Best, J. , Parker, S. , Rostrup, N. , & Stanley, E. A. (2015). Minds “at attention”: Mindfulness training curbs attentional lapses in military cohorts. PLoS One, 10, e0116889. 25671579 10.1371/journal.pone.0116889 PMC4324839

[hbm25197-bib-0041] Jha, A. P. , Stanley, E. A. , Kiyonaga, A. , Wong, L. , & Gelfand, L. (2010). Examining the protective effects of mindfulness training on working memory capacity and affective experience. Emotion, 10, 54–64. 20141302 10.1037/a0018438

[hbm25197-bib-0042] Keller, J. B. , Hedden, T. , Thompson, T. W. , Anteraper, S. A. , Gabrieli, J. D. E. , & Whitfield‐Gabrieli, S. (2015). Resting‐state anticorrelations between medial and lateral prefrontal cortex: Association with working memory, aging, and individual differences. Cortex, 64, 271–280. 25562175 10.1016/j.cortex.2014.12.001 PMC4346444

[hbm25197-bib-0043] Kelly, A. M. C. , Clare Kelly, A. M. , Uddin, L. Q. , Biswal, B. B. , Xavier Castellanos, F. , & Milham, M. P. (2008). Competition between functional brain networks mediates behavioral variability. NeuroImage, 39, 527–537. 17919929 10.1016/j.neuroimage.2007.08.008

[hbm25197-bib-0044] Lawler, J. M. , Esposito, E. A. , Doyle, C. M. , & Gunnar, M. R. (2019). A preliminary, randomized‐controlled trial of mindfulness and game‐based executive function trainings to promote self‐regulation in internationally‐adopted children. Development and Psychopathology, 31(4), 1513–1525. 30698120 10.1017/S0954579418001190

[hbm25197-bib-0045] Leonard, N. R. , Jha, A. P. , Casarjian, B. , Goolsarran, M. , Garcia, C. , Cleland, C. M. , … Massey, Z. (2013). Mindfulness training improves attentional task performance in incarcerated youth: A group randomized controlled intervention trial. Frontiers in Psychology, 4, 792. 24265621 10.3389/fpsyg.2013.00792 PMC3820955

[hbm25197-bib-0046] Lutz, A. , Brefczynski‐Lewis, J. , Johnstone, T. , & Davidson, R. J. (2008). Regulation of the neural circuitry of emotion by compassion meditation: Effects of meditative expertise. PLoS One, 3, e1897. 18365029 10.1371/journal.pone.0001897 PMC2267490

[hbm25197-bib-0047] Lutz, A. , Dunne, J. D. , & Davidson, R. J. (2007). Meditation and the neuroscience of consciousness In Zelazo P., Moscovitch M., & Thompson E. (Eds.), Cambridge handbook of consciousness, (499–551). Cambridge, England: Cambridge University Press.

[hbm25197-bib-0048] Lutz, A. , Slagter, H. A. , Dunne, J. D. , & Davidson, R. J. (2008). Attention regulation and monitoring in meditation. Trends in Cognitive Sciences, 12, 163–169. 18329323 10.1016/j.tics.2008.01.005 PMC2693206

[hbm25197-bib-0049] Mak, C. , Whittingham, K. , Cunnington, R. , & Boyd, R. N. (2018). Efficacy of mindfulness‐based interventions for attention and executive function in children and adolescents—A systematic review. Mindfulness, 9, 59–78.

[hbm25197-bib-0050] Mason, M. F. , Norton, M. I. , van Horn, J. D. , Wegner, D. M. , Grafton, S. T. , & Macrae, C. N. (2007). Wandering minds: The default network and stimulus‐independent thought. Science, 315, 393–395. 17234951 10.1126/science.1131295 PMC1821121

[hbm25197-bib-0051] Mattfeld, A. T. , Gabrieli, J. D. E. , Biederman, J. , Spencer, T. , Brown, A. , Kotte, A. , … Whitfield‐Gabrieli, S. (2014). Brain differences between persistent and remitted attention deficit hyperactivity disorder. Brain, 137, 2423–2428. 24916335 10.1093/brain/awu137

[hbm25197-bib-0052] McVay, J. C. , & Kane, M. J. (2009). Conducting the train of thought: Working memory capacity, goal neglect, and mind wandering in an executive‐control task. Journal of Experimental Psychology. Learning, Memory, and Cognition, 35, 196–204. 10.1037/a0014104 PMC2750806 19210090

[hbm25197-bib-0053] Moffitt, T. E. , Arseneault, L. , Belsky, D. , Dickson, N. , Hancox, R. J. , Harrington, H. , … Caspi, A. (2011). A gradient of childhood self‐control predicts health, wealth, and public safety. Proceedings of the National Academy of Sciences of the United States of America, 108, 2693–2698. 21262822 10.1073/pnas.1010076108 PMC3041102

[hbm25197-bib-0054] Moore, A. , & Malinowski, P. (2009). Meditation, mindfulness and cognitive flexibility. Consciousness and Cognition, 18, 176–186. 19181542 10.1016/j.concog.2008.12.008

[hbm25197-bib-0055] Morgan, K. L. , & Rubin, D. B. (2012). Rerandomization to improve covariate balance in experiments. The Annals of Statistics, 40, 1263–1282.

[hbm25197-bib-0056] Muris, P. (2006). Relation of attention control and school performance in normal children. Perceptual and Motor Skills, 102, 78–80. 16671601 10.2466/pms.102.1.78-80

[hbm25197-bib-0057] Ng, K. K. , Lo, J. C. , Lim, J. K. W. , Chee, M. W. L. , & Zhou, J. (2016). Reduced functional segregation between the default mode network and the executive control network in healthy older adults: A longitudinal study. NeuroImage, 133, 321–330. 27001500 10.1016/j.neuroimage.2016.03.029

[hbm25197-bib-0058] Oldfield, R. C. (1971). The assessment and analysis of handedness: The Edinburgh inventory. Neuropsychologia, 9, 97–113. 5146491 10.1016/0028-3932(71)90067-4

[hbm25197-bib-0059] Paus, T. , Keshavan, M. , & Giedd, J. N. (2008). Why do many psychiatric disorders emerge during adolescence? Nature Reviews. Neuroscience, 9, 947–957. 19002191 10.1038/nrn2513 PMC2762785

[hbm25197-bib-0060] Peirce, J. W. (2007). PsychoPy—Psychophysics software in Python. Journal of Neuroscience Methods, 162, 8–13. 17254636 10.1016/j.jneumeth.2006.11.017 PMC2018741

[hbm25197-bib-0061] Piccoli, T. , Valente, G. , Linden, D. E. J. , Re, M. , Esposito, F. , Sack, A. T. , & di Salle, F. (2015). The default mode network and the working memory network are not anti‐correlated during all phases of a working memory task. PLoS One, 10, e0123354. 25848951 10.1371/journal.pone.0123354 PMC4388669

[hbm25197-bib-0062] Polak, E. L. (2009). Impact of Two Sessions of Mindfulness Training on Attention. University of Miami. Retrieved from https://scholarlyrepository.miami.edu/cgi/viewcontent.cgi?referer=https://scholar.google.com/&httpsredir=1&article=1250&context=oa_dissertations.

[hbm25197-bib-0063] Poldrack, R. A. (2007). Region of interest analysis for fMRI. Social Cognitive and Affective Neuroscience, 2, 67–70. 18985121 10.1093/scan/nsm006 PMC2555436

[hbm25197-bib-0064] Posner, J. , Park, C. , & Wang, Z. (2014). Connecting the dots: A review of resting connectivity MRI studies in attention‐deficit/hyperactivity disorder. Neuropsychology Review, 24, 3–15. 24496902 10.1007/s11065-014-9251-z PMC4119002

[hbm25197-bib-0065] Quach, D. , Jastrowski Mano, K. E. , & Alexander, K. (2016). A randomized controlled trial examining the effect of mindfulness meditation on working memory capacity in adolescents. The Journal of Adolescent Health, 58, 489–496. 26576819 10.1016/j.jadohealth.2015.09.024

[hbm25197-bib-0066] Rabiner, D. , & Coie, J. D. (2000). Early attention problems and children's reading achievement: A longitudinal investigation. Journal of the American Academy of Child and Adolescent Psychiatry, 39, 859–867. 10892227 10.1097/00004583-200007000-00014 PMC2777533

[hbm25197-bib-0067] Robertson, I. H. , Manly, T. , Andrade, J. , Baddeley, B. T. , & Yiend, J. (1997a). Oops!': Performance correlates of everyday attentional failures in traumatic brain injured and normal subjects. Neuropsychologia, 35, 747–758. 9204482 10.1016/s0028-3932(97)00015-8

[hbm25197-bib-0068] Robertson, I. H. , Manly, T. , Andrade, J. , Baddeley, B. T. , & Yiend, J. (1997b). Sustained attention to response task, Ann Arbor, MI: ProQuest PsycTESTS Dataset 10.1037/t28308-000

[hbm25197-bib-0069] R‐Project. R Core Team . (2014). R: A language and environment for statistical computing. Vienna, Austria: R Foundation for Statistical Computing Retrieved from http://www.R-project.org/

[hbm25197-bib-0070] Rudolph, M. D. , Miranda‐Domínguez, O. , Cohen, A. O. , Breiner, K. , Steinberg, L. , Bonnie, R. J. , … Fair, D. A. (2017). At risk of being risky: The relationship between “brain age” under emotional states and risk preference. Developmental Cognitive Neuroscience, 24, 93–106. 28279917 10.1016/j.dcn.2017.01.010 PMC5849238

[hbm25197-bib-0071] Salmoirago‐Blotcher, E. , Druker, S. , Meleo‐Meyer, F. , Frisard, C. , Crawford, S. , & Pbert, L. (2019). Beneficial effects of school‐based mindfulness training on impulsivity in healthy adolescents: Results from a pilot randomized controlled trial. EXPLORE, 15, 160–164. 30309789 10.1016/j.explore.2018.07.003 PMC9678328

[hbm25197-bib-0072] Sarter, M. , Givens, B. , & Bruno, J. P. (2001). The cognitive neuroscience of sustained attention: Where top‐down meets bottom‐up. Brain Research Reviews, 35, 146–160. 11336780 10.1016/s0165-0173(01)00044-3

[hbm25197-bib-0073] Schonert‐Reichl, K. A. , Oberle, E. , Lawlor, M. S. , Abbott, D. , Thomson, K. , Oberlander, T. F. , & Diamond, A. (2015). Enhancing cognitive and social–emotional development through a simple‐to‐administer mindfulness‐based school program for elementary school children: A randomized controlled trial. Developmental Psychology, 51, 52–66. 25546595 10.1037/a0038454 PMC4323355

[hbm25197-bib-0074] SCRATCH . (n.d.). Retrieved from https://scratch.mit.edu/.

[hbm25197-bib-0075] Semple, R. J. , Lee, J. , Rosa, D. , & Miller, L. F. (2010). A randomized trial of mindfulness‐based cognitive therapy for children: Promoting mindful attention to enhance social‐emotional resiliency in children. Journal of Child and Family Studies, 19, 218–229.

[hbm25197-bib-0076] Sherman, L. E. , Rudie, J. D. , Pfeifer, J. H. , Masten, C. L. , McNealy, K. , & Dapretto, M. (2014). Development of the default mode and central executive networks across early adolescence: A longitudinal study. Developmental Cognitive Neuroscience, 10, 148–159. 25282602 10.1016/j.dcn.2014.08.002 PMC4854607

[hbm25197-bib-0077] Sidhu, P. (2012). *The efficacy of mindfulness meditation in increasing the attention span in children with ADHD* Groth‐Marnat G. (PhD thesis). Pacifica Graduate Institute. Retrieved from https://books.google.com/books/about/The_Efficacy_of_Mindfulness_Meditation_i.html?hl=&id=l_53oAEACAAJ.

[hbm25197-bib-0078] Smallwood, J. (2013). Penetrating the fog of the decoupled mind: The effects of visual salience in the sustained attention to response task. Canadian Journal of Experimental Psychology, 67, 32–40. 23458549 10.1037/a0030760

[hbm25197-bib-0079] Spira, E. G. , & Fischel, J. E. (2005). The impact of preschool inattention, hyperactivity, and impulsivity on social and academic development: A review. Canadian Journal of Psychology, 46, 755–773. 10.1111/j.1469-7610.2005.01466.x 15972069

[hbm25197-bib-0080] Taren, A. A. , Gianaros, P. J. , Greco, C. M. , Lindsay, E. K. , Fairgrieve, A. , Brown, K. W. , … Creswell, J. D. (2015). Mindfulness meditation training alters stress‐related amygdala resting state functional connectivity: A randomized controlled trial. Social Cognitive and Affective Neuroscience, 10, 1758–1768. 26048176 10.1093/scan/nsv066 PMC4666115

[hbm25197-bib-0081] Taren, A. A. , Gianaros, P. J. , Greco, C. M. , Lindsay, E. K. , Fairgrieve, A. , Brown, K. W. , … Creswell, J. D. (2017). Mindfulness meditation training and executive control network resting state functional connectivity: A randomized controlled trial. Psychosomatic Medicine, 79, 674–683. 28323668 10.1097/PSY.0000000000000466 PMC5489372

[hbm25197-bib-0082] Thesen, S. , Heid, O. , Mueller, E. , & Schad, L. R. (2000). Prospective acquisition correction for head motion with image‐based tracking for real‐time fMRI. Magnetic Resonance in Medicine, 44, 457–465. 10975899 10.1002/1522-2594(200009)44:3<457::aid-mrm17>3.0.co;2-r

[hbm25197-bib-0083] van Dijk, K. R. A. , Hedden, T. , Venkataraman, A. , Evans, K. C. , Lazar, S. W. , & Buckner, R. L. (2010). Intrinsic functional connectivity as a tool for human connectomics: Theory, properties, and optimization. Journal of Neurophysiology, 103, 297–321. 19889849 10.1152/jn.00783.2009 PMC2807224

[hbm25197-bib-0084] Wechsler, D. (1999). Wechsler Abbreviated Scale of Intelligence, Ann Arbor, MI: ProQuest PsycTESTS Dataset 10.1037/t15170-000

[hbm25197-bib-0085] Weissman, D. H. , Roberts, K. C. , Visscher, K. M. , & Woldorff, M. G. (2006). The neural bases of momentary lapses in attention. Nature Neuroscience, 9, 971–978. 16767087 10.1038/nn1727

[hbm25197-bib-0086] Whitfield‐Gabrieli, S. , Fischer, A. S. , Henricks, A. M. , Khokhar, J. Y. , Roth, R. M. , Brunette, M. F. , & Green, A. I. (2018). Understanding marijuana's effects on functional connectivity of the default mode network in patients with schizophrenia and co‐occurring cannabis use disorder: A pilot investigation. Schizophrenia Research, 194, 70–77. 28823723 10.1016/j.schres.2017.07.029 PMC6886576

[hbm25197-bib-0087] Whitfield‐Gabrieli, S. , & Nieto‐Castanon, A. (2012). Conn: A functional connectivity toolbox for correlated and anticorrelated brain networks. Brain Connectivity, 2, 125–141. 22642651 10.1089/brain.2012.0073

[hbm25197-bib-0088] Whitfield‐Gabrieli, S. , Thermenos, H. W. , Milanovic, S. , Tsuang, M. T. , Faraone, S. V. , McCarley, R. W. , … Seidman, L. J. (2009). Hyperactivity and hyperconnectivity of the default network in schizophrenia and in first‐degree relatives of persons with schizophrenia. Proceedings of the National Academy of Sciences of the United States of America, 106, 1279–1284. 19164577 10.1073/pnas.0809141106 PMC2633557

[hbm25197-bib-0089] Wilson, B. J. , Petaja, H. , & Mancil, L. (2011). The attention skills and academic performance of aggressive/rejected and low aggressive/popular children. Early Education and Development, 22, 907–930.

[hbm25197-bib-0090] Woods, D. L. , & Knight, R. T. (1986). Electrophysiologic evidence of increased distractibility after dorsolateral prefrontal lesions. Neurology, 36, 212–216. 3945393 10.1212/wnl.36.2.212

[hbm25197-bib-0091] Worsley, K. J. , Marrett, S. , Neelin, P. , Vandal, A. C. , Friston, K. J. , & Evans, A. C. (1996). A unified statistical approach for determining significant signals in images of cerebral activation. Human Brain Mapping, 4, 58–73. 20408186 10.1002/(SICI)1097-0193(1996)4:1<58::AID-HBM4>3.0.CO;2-O

[hbm25197-bib-0092] Yang, C.‐C. , Barrós‐Loscertales, A. , Pinazo, D. , Ventura‐Campos, N. , Borchardt, V. , Bustamante, J.‐C. , … Walter, M. (2016). State and training effects of mindfulness meditation on brain networks reflect neuronal mechanisms of its antidepressant effect. Neural Plasticity, 2016, 9504642. 26998365 10.1155/2016/9504642 PMC4779536

[hbm25197-bib-0093] Yeager, D. S. , & Dweck, C. S. (2012). Mindsets that promote resilience: When students believe that personal characteristics can be developed. Educational Psychologist, 47, 302–314.

