# Supplementary material for: Mindfulness training preserves sustained attention and resting state anticorrelation between default‐mode network and dorsolateral prefrontal cortex: A randomized controlled trial
Source: Hum Brain Mapp. 2020 Sep 24;41(18):5356–69. doi: 10.1002/hbm.25197 (PMC7670646; doi:10.1002/hbm.25197)
Supplement: Supplementary file 1 — Figure S1.1 Residual correlation across subjects between Functional Connectivity (FC) and a Quality Control measure (QC) indicative of average subject motion, before (top) and after (bottom) denoising of the BOLD signal. Figure S1.2. Distributions of functional connectivity values (bivariate correlation coefficients) in a network of 512 nodes across the entire brain, computed separately for each subject during pre‐ (top) and post‐ (bottom) sessions. [file HBM-41-5356-s001.docx]

### **Supplementary Material**

### **S1.0 Motion correction effectiveness analyses**

###

### S1.01 Distribution of Quality Control-Functional Connectivity Associations (QC-FC associations) for mean motion. These analyses (see Power et al. 2014 and Ciric et al. 2017 for details) show the correlations across-subject between subject motion (scan-to-scan Framewise Displacement averaged across the entire session) and functional connectivity (bivariate correlation coefficients), computed separately for each pair of nodes in a network of 512 randomly distributed locations across the entire brain. The resulting distribution of motion-connectivity correlations (showed as grayed area in Figure S1.1) is contrasted with the expected distribution of the same measure under the null hypothesis (shown by a red dashed line, and estimated using random permutation non-parametric analyses). The results indicate low absolute mean motion-connectivity correlations after denoising (r=0.01, comparable to many of the best approaches analyzed in Ciric et al. 2017), as well as correlation distributions very similar to those expected purely by chance (96.7% Kolmogorov-Smirnov match after denoising, above the 95% threshold recommended in Nieto-Castanon 2020). In addition, denoising correctly centers the distribution of functional connectivity values (Figure S1.2), with global correlation (GCOR, average functional connectivity among the 512 nodes described above) changing from GCOR=0.25±0.09 and GCOR=0.28±0.10 before denoising in pre- and post- sessions, respectively, to GCOR=0.04±0.04 and GCOR=0.02±0.02 after denoising.

### Combined, these analyses suggest that the preprocessing pipeline used in the present analysis was effective in reducing artifactual biases in FC estimates while minimizing any residual association between subject motion and functional connectivity.

### Figure S1.1. Residual correlation across subjects between Functional Connectivity (FC) and a Quality Control measure (QC) indicative of average subject motion, before (top) and after (bottom) denoising of the BOLD signal.
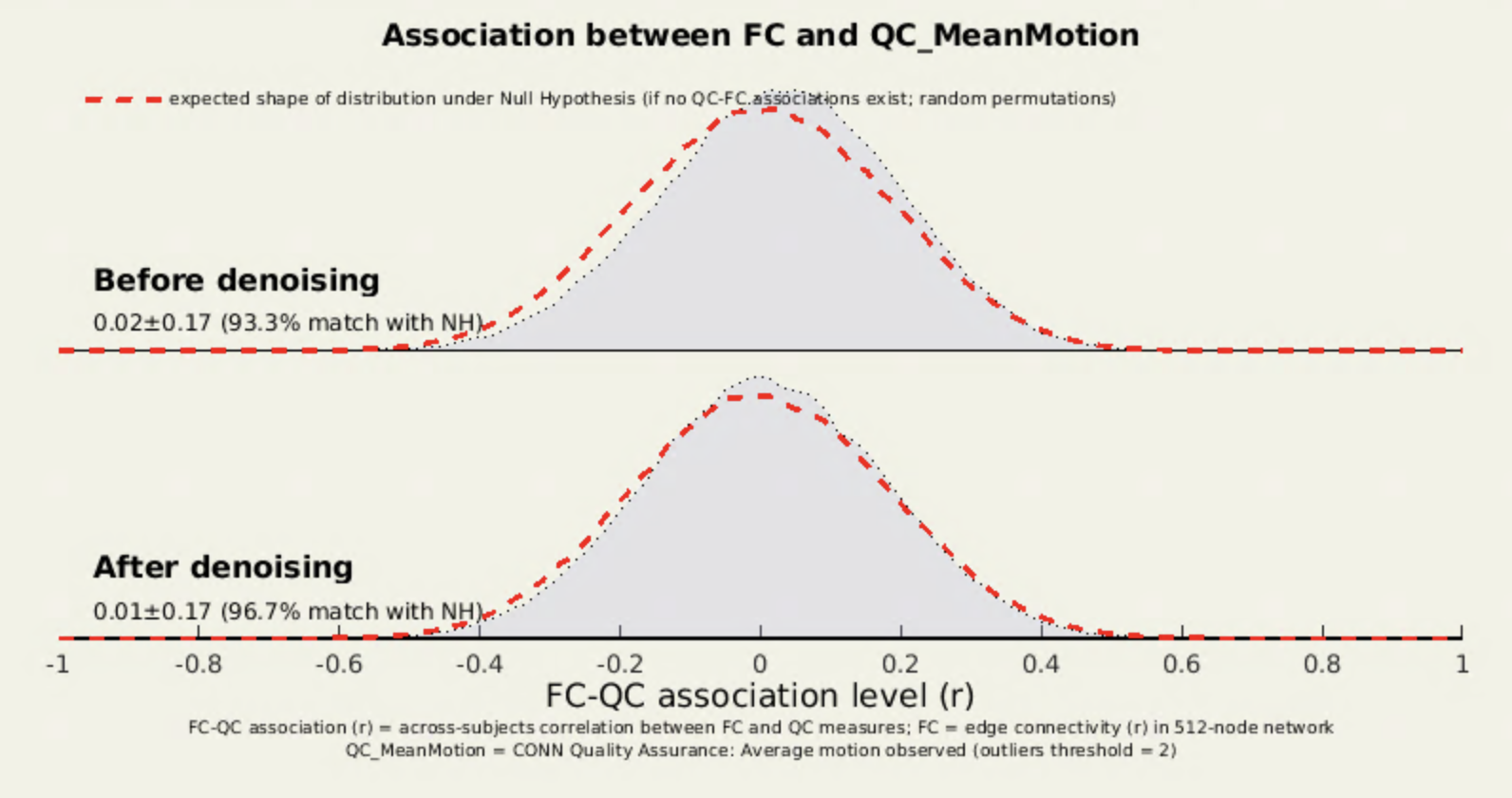


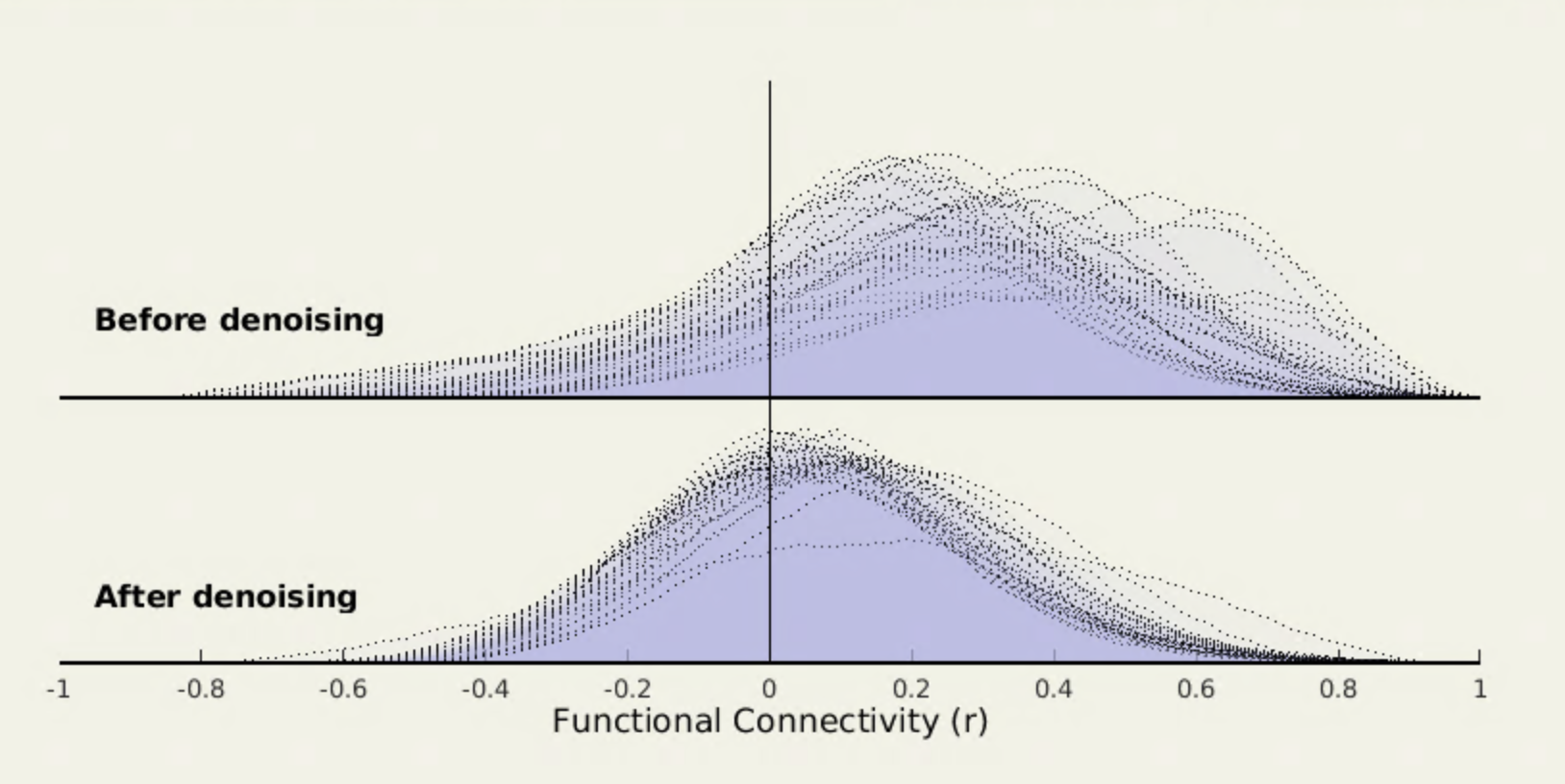


###
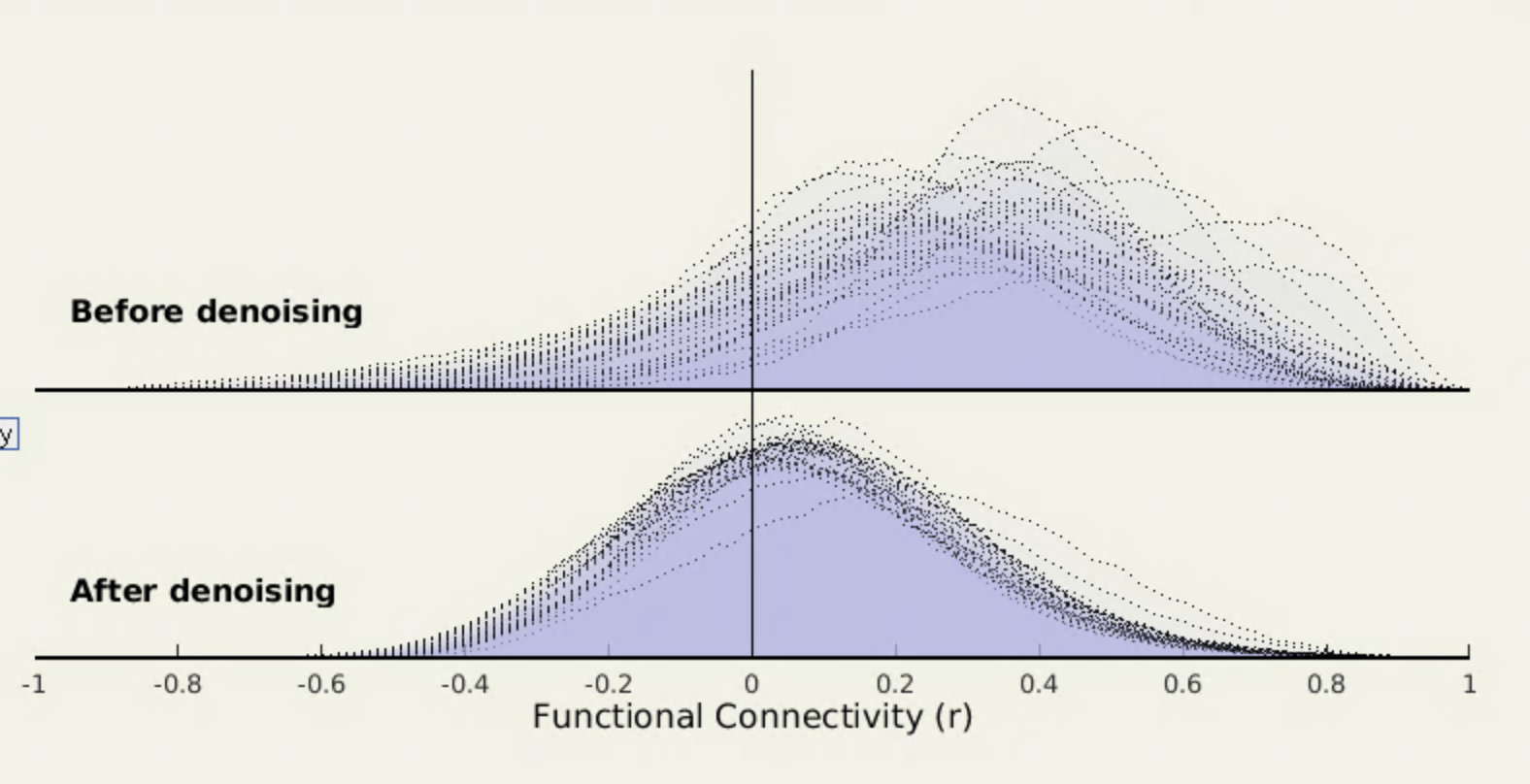


*Figure S1.2. Distributions of functional connectivity values (bivariate correlation coefficients) in a network of 512 nodes across the entire brain, computed separately for each subject during pre- (top) and post- (bottom) sessions.*

###

### REFERENCES

###

### Ciric, R., Wolf, D. H., Power, J. D., Roalf, D. R., Baum, G. L., Ruparel, K., … & Gur, R. C. (2017). Benchmarking of participant-level confound regression strategies for the control of motion artifact in studies of functional connectivity. Neuroimage, 154, 174-187.

Nieto-Castanon, A. (2020). Handbook of fcMRI methods in CONN. Boston, MA: Hilbert Press

Power, J. D., Mitra, A., Laumann, T. O., Snyder, A. Z., Schlaggar, B. L., & Petersen, S. E. (2014). Methods to detect, characterize, and remove motion artifact in resting state fMRI. Neuroimage, 84, 320-341.
